# Structure of an open K_ATP_ channel reveals tandem PIP_2_ binding sites mediating the Kir6.2 and SUR1 regulatory interface

**DOI:** 10.1038/s41467-024-46751-5

**Published:** 2024-03-20

**Authors:** Camden M. Driggers, Yi-Ying Kuo, Phillip Zhu, Assmaa ElSheikh, Show-Ling Shyng

**Affiliations:** 1 Department of Chemical Physiology and Biochemistry, School of Medicine, Oregon Health & Science University, Portland, OR 97239 USA; 2 Department of Medical Biochemistry, Tanta University, Tanta, Egypt

**Keywords:** Cryoelectron microscopy, Potassium channels, Permeation and transport

## Abstract

ATP-sensitive potassium (K_ATP_) channels, composed of four pore-lining Kir6.2 subunits and four regulatory sulfonylurea receptor 1 (SUR1) subunits, control insulin secretion in pancreatic β-cells. K_ATP_ channel opening is stimulated by PIP_2_ and inhibited by ATP. Mutations that increase channel opening by PIP_2_ reduce ATP inhibition and cause neonatal diabetes. Although considerable evidence has implicated a role for PIP_2_ in K_ATP_ channel function, previously solved open-channel structures have lacked bound PIP_2_, and mechanisms by which PIP_2_ regulates K_ATP_ channels remain unresolved. Here, we report the cryoEM structure of a K_ATP_ channel harboring the neonatal diabetes mutation Kir6.2-Q52R, in the open conformation, bound to amphipathic molecules consistent with natural C18:0/C20:4 long-chain PI(4,5)P_2_ at two adjacent binding sites between SUR1 and Kir6.2. The canonical PIP_2_ binding site is conserved among PIP_2_-gated Kir channels. The non-canonical PIP_2_ binding site forms at the interface of Kir6.2 and SUR1. Functional studies demonstrate both binding sites determine channel activity. Kir6.2 pore opening is associated with a twist of the Kir6.2 cytoplasmic domain and a rotation of the N-terminal transmembrane domain of SUR1, which widens the inhibitory ATP binding pocket to disfavor ATP binding. The open conformation is particularly stabilized by the Kir6.2-Q52R residue through cation-π bonding with SUR1-W51. Together, these results uncover the cooperation between SUR1 and Kir6.2 in PIP_2_ binding and gating, explain the antagonistic regulation of K_ATP_ channels by PIP_2_ and ATP, and provide a putative mechanism by which Kir6.2-Q52R stabilizes an open channel to cause neonatal diabetes.

## Abstract (web summary)

K_ATP_ channels regulate insulin secretion and are activated by PIP_2_. Here, the authors show PIP_2_ binds between SUR1 and Kir6.2 to open the channel, and a neonatal diabetes mutation stabilizes K_ATP_ channels in a PIP_2_-bound open conformation.

## Introduction

The K_ATP_ channel expressed in pancreatic islet β-cells is a principal homeostatic regulator of insulin secretion^1–3^. Composed of four pore-forming Kir6.2 subunits and four regulatory sulfonylurea receptor 1 (SUR1) subunits^4,5^, K_ATP_ channels influence the resting membrane potential, thus action potential firing, Ca^2+^ entry, and insulin secretion. The activity of K_ATP_ channels is dynamically regulated by glucose changes via intracellular ATP and MgADP, which bind to inhibitory and stimulatory sites to close and open the channel, respectively^6,7^. This enables insulin secretion to follow fluctuations in glucose concentrations. The central role of K_ATP_ channels in insulin secretion and glucose homeostasis is underscored by dysregulated insulin secretion and blood glucose in humans bearing K_ATP_ mutations: loss-of-function mutations cause congenital hyperinsulinism characterized by persistent insulin secretion despite life-threatening hypoglycemia^8,9^; conversely, gain-of-function channel mutations result in neonatal diabetes due to insufficient insulin secretion^10^.

In addition to adenine nucleotides-dependent regulation, membrane phosphoinositides participate in the gating activation of K_ATP_ channels^11,12^, as they do all other eukaryotic Kir channels^13^. PI(4,5)P_2_ (denoted as PIP_2_ unless specified) is the most abundant phosphoinositide in the plasma membrane^14^, with PIP_2_ containing arachidonoyl (C20:4) and stearoyl (C18:0) acyl chains being the most dominant species in mammalian cells^15^. In inside-out patch-clamp recordings, application of C18:0/C20:4 long-chain PIP_2_ to the cytoplasmic side of the membrane patch resulted in stable increases in K_ATP_ channel open probability (*P*_*o*_) and a gradual decrease in channel sensitivity to ATP inhibition; whereas scavenging membrane PIP_2_ with poly-lysine decreases channel *P*_*o*_^11,12^. Because increasing channel *P*_*o*_ by PIP_2_ also decreases channel sensitivity to ATP inhibition^11,12^, a joint regulatory mechanism involving allosteric or binding competition between the two ligands has seemed likely^16^. In particular, the effect of PIP_2_ on K_ATP_ channels has been assumed to derive from a putative conserved binding site in Kir6.2 having a position homologous to the PIP_2_ binding site previously identified in crystal and cryoEM structures of Kir2 and Kir3^17–20^. Unlike other Kir channels however, gating of Kir6.2 by PIP_2_ is strongly modulated by its regulatory subunit SUR1. Compared to the fully assembled K_ATP_ channels, Kir6.2 channels lacking SUR1 partners display more than 10-fold lower *P*_*o*_^11,16,21^, which reflects reduced PIP_2_ binding or gating, and are less sensitive to PIP_2_ stimulation^11,16^. Of particular interest, the SUR1 N-terminal transmembrane domain, TMD0, which interfaces with Kir6.2^22–24^, is sufficient to confer the high *P*_*o*_ of wild-type (WT) channels^25,26^, implicating a crucial role of SUR1-TMD0 in controlling Kir6.2 response to PIP_2_.

To date, cryoEM structures of K_ATP_ channels determined for a variety of liganded and mutational states have yielded a structural spectrum encompassing open, closed, and inactivated conformations (reviewed in^6,7,27^). Careful comparisons have suggested mechanisms by which channel activity is regulated by the SUR subunit and nucleotides. However, confusion has grown regarding PIP_2_ binding and gating. Two recently reported open- and pre-open mutant K_ATP_ channel cryoEM structures lack discernible PIP_2_ at the putative binding site^28,29^, even when excess synthetic short-chain diC8-PI(4,5)P_2_ was added to the sample in the case of the pre-open structure^30^. Furthermore, purified WT K_ATP_ channels reconstituted in lipid bilayers lacking PIP_2_ showed single channel openings^28^. These reports suggest that PIP_2_ binding may be inessential for open channel transition^28^. Thus, notwithstanding robust data indicating PIP_2_ involvement in physiological channel activation^11,31,32^, how PIP_2_ binds and modulates K_ATP_ channels remains enigmatic.

Our approach to understanding K_ATP_ channel activation and PIP_2_ binding involved determining channel structure bound to PIP_2_ by (1) incubating membranes expressing K_ATP_ channels with long-chain PIP_2_ known to stably activate the channel^11,12,33^, and (2) employing a Kir6.2 variant containing the neonatal diabetes-causing mutation Q52R (denoted Kir6.2^Q52R^)^34,35^. Kir6.2^Q52R^ markedly increases channel *P*_*o*_ and causes a 20-fold decrease in channel sensitivity to ATP inhibition^34,35^. Higher *P*_*o*_ could result from enhanced PIP_2_ binding or its effect on gating. Intriguingly, the effect of this mutation on channel gating is SUR1-dependent. When Kir6.2 was manipulated to express at the cell surface without co-assembly with SUR1 (by deleting the Arg-Lys-Arg endoplasmic reticulum retention signal at the C-terminal region of Kir6.2), introducing the Q52R mutation had little effect on the *P*_*o*_ and ATP sensitivity^36^. The SUR1-dependent overactivity imparted by the Kir6.2-Q52R mutation indicated the possibility to probe the regulatory interaction between SUR1 and membrane PIP_2_ on Kir6.2 gating.

Here, we present the cryoEM structure of the K_ATP_ channel harboring the neonatal diabetes Q52R mutation (hereinafter referred to as the SUR1/Kir6.2^Q52R^ channel) in an open conformation bound to amphipathic molecules consistent with natural C18:0/C20:4 long-chain PI(4,5)P_2_. Surprisingly, our structural model suggests that two PIP_2_ molecules bind in tandem in the open channel at the interface between Kir6.2 and SUR1. At each Kir6.2-SUR1 interface, one PIP_2_ molecule is bound within the site that is conserved among Kir channels (referred to as the canonical site), and a second PIP_2_ molecule is at an adjacent site, stacked within the regulatory interface between Kir6.2 and SUR1-TMD0 (referred to as the non-canonical site). The open channel conformation is further stabilized by direct interaction between Q52R of Kir6.2 and W51 located in TMD0 of SUR1. Accounting for decades of evidence from functional perturbation studies, the PIP_2_-bound SUR1/Kir6.2^Q52R^ K_ATP_ structure unveils the agonist binding of PIP_2_ in K_ATP_ channels, revealing that in addition to the ancestral Kir PIP_2_ site, the incorporation of the regulatory SUR1 subunit creates a second regulatory PIP_2_ binding site. The results uncover a mechanism through which SUR1, by cooperating with Kir6.2 for PIP_2_ binding, stabilizes the K_ATP_ channel in an open state to control activity, and illuminate a putative molecular mechanism by which a neonatal diabetes-associated Kir6.2 mutation causes overactive K_ATP_ channels.

## Results

### Structure of an open SUR1/Kir6.2^Q52R^ K_ATP_ channel bound to PIP_2_

To purify the neonatal diabetes variant SUR1/Kir6.2^Q52R^ K_ATP_ channel, we co-expressed Kir6.2^Q52R^ and SUR1 as independent polypeptides by transducing adherent mammalian COSm6 cells with recombinant adenovirus packaged with genes for rat Kir6.2^Q52R^ and FLAG-tagged hamster SUR1 (see Methods). Prior functional experiments have shown that the activating effects of long-chain PIP_2_ are long-lasting and resistant to subsequent washout, indicating stable partition into the membrane^11,12,33^. By contrast, the effects of synthetic short-chain PIP_2_ such as diC8-PIP_2_ are highly reversible^33,37^. Accordingly, we prepared a membrane fraction of COSm6 cells expressing the mutant channel, and enriched the native membranes with brain PIP_2_, primarily containing C18:0/C20:4 long-chain PI(4,5)P_2_ (Avanti Polar Lipids), before detergent solubilization. We anticipated that the addition of long-chain PIP_2_ prior to detergent extraction would stably incorporate PIP_2_ resistant to washout in subsequent purification steps to facilitate the capture of PIP_2_-bound channel structure. K_ATP_ channels were purified via the N-terminal FLAG-tag on SUR1 and spotted on graphene oxide (GO)-coated grids for cryoEM (see Methods). To maximize channels in PIP_2_-bound open conformations, no ATP or ADP were added to the sample.

**Fig. 1:**
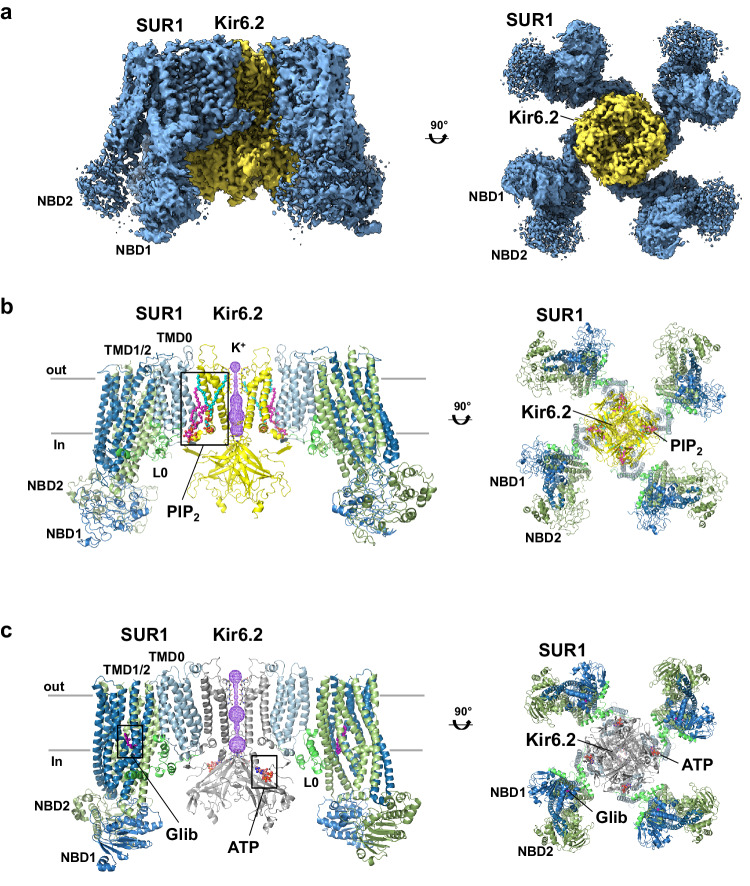
CryoEM structure of PIP_2_-bound, open SUR1/Kir6.2^Q52R^ K_ATP_ channel. **a** The 2.9 Å cryoEM map (the micelle not shown), from C4 non-uniform refinement of 14,115 particles, shown from the side and bottom (cytoplasmic view). **b** Structural model of the SUR1/Kir6.2^Q52R^ K_ATP_ channel in complex with PIP_2_. The potassium ion selectivity filter is shown as sticks with oxygens colored red, the K^+^ conduction pathway is shown as purple mesh. Only two Kir6.2 and two SUR1 subunits are shown in the side view for clarity. A view rotated 90° (cytoplasmic view) displays all four Kir6.2 subunits and all four SUR1 subunits. **c** Structural model of a closed WT SUR1/Kir6.2 K_ATP_ channel in complex with ATP and gliblenclamide (Glib) (PDB ID 6BAA), shown for comparison and rendered in PyMOL similar to (**b**), except for the Kir6.2 subunits which are colored gray.

CryoEM micrographs showed mostly full SUR1/Kir6.2^Q52R^ K_ATP_ channel particles (Supplementary Fig. 1a). 2D classes indicate an ordered Kir6.2 and SUR1-TMD0, with a relatively more blurred SUR1-ABC core especially the nucleotide-binding domains (NBDs) (Supplementary Fig. 1a–d). A non-uniform refinement with C4 symmetry using all good particles from 2D classification (70,638) in cryoSPARC resulted in an un-masked reconstruction of the full channel at 3.9 Å using the gold-standard Fourier shell correlation (GSFSC) cutoff of 0.143 (see Methods). The electron potential map shows a relatively weak signal for TMD2 and NBD2 of SUR1 (Supplementary Fig. 1), indicating higher mobility of these domains. Multiple rounds of ab initio reconstruction without symmetry imposed (Supplementary Fig. 1a) yielded a best 3D class containing 14,115 particles, which following a C4 non-uniform refinement in cryoSPARC with auto-masking that excluded SUR1-NBD2 resulted in a final map reconstruction of 2.9 Å by GSFSC within the autoFSC mask (Fig.1a, Supplementary Fig. 1a–d), or 3.3 Å resolution by GSFSC within a mask that includes the full K_ATP_ channel. Since the full channel map derived from the C4 non-uniform refinement has the best overall resolution, showing a highly ordered Kir6.2 tetramer core and SUR1-TMD0 with local resolutions < 2.9 Å, the map was used for model building (Fig.1, Supplementary Fig. 1, Supplementary Table 1). The cryoEM map reconstruction revealed amphipathic densities that are fit by two bound PIP_2_ molecules at the regulatory interface of Kir6.2 and SUR1, an open Kir6.2 pore, and molecular rearrangements at the SUR1-Kir6.2 interface critical to gating, as we describe in detail in the following sections.

Additional analysis of all particles from the 2D classes employed a strategy of symmetry expansion and focused 3D classification (using a mask of Kir6.2^Q52R^ tetramer plus one SUR1 subunit to circumvent the flexibility of the SUR1 subunits), revealing a spectrum of conformational classes with distinct Kir6.2-cytoplasmic domain (CTD) and SUR1 positions (Supplementary Fig. 2). The two most extreme conformations include a dominant conformation at ~3.3 Å (no C4 symmetry imposed) that is nearly identical to the map derived from the C4 non-uniform refinement described above, and a minor conformation at 6.9 Å (Supplementary Fig. 2a). Superimposition of the two extreme conformations revealed differences in the K^+^ pore path, rotation of the Kir6.2^Q52R^-CTD, and distance of the Kir6.2^Q52R^-CTD to the membrane (Supplementary Fig. 2b–d), as well as the absence or presence of the cryoEM density corresponding to the N-terminal domain of Kir6.2^Q52R^ (the distal 30 amino acids, referred to as KNtp; Supplementary Fig. 2e). Relative to the high resolution dominant class, the low resolution minor class shows a constricted Kir6.2^Q52R^ pore, a clockwise-rotated (viewed from the cytoplasmic side) Kir6.2^Q52R^-CTD that is moved away from the membrane and into the cytoplasm, and a clear KNtp density in the central cleft of the SUR1-ABC core. Moreover, the SUR1-ABC core is rotated towards the Kir6.2^Q52R^ tetramer and moved downward from the outer membrane (Supplementary Fig. 2c, d). These features highly resemble our previously published closed apo (no PIP_2_, nucleotides, or pharmacological inhibitors added) WT K_ATP_ channel structure (PDB ID 7UQR)^38^, and indicate the minor conformation isolated from our sample represents an apo closed SUR1/Kir6.2^Q52R^ structure (and will be referred to as such hereinafter) even with the exogenously added long-chain PIP_2_.

### Tandem PIP_2_ binding at the interface of Kir6.2 and SUR1

**Fig. 2:**
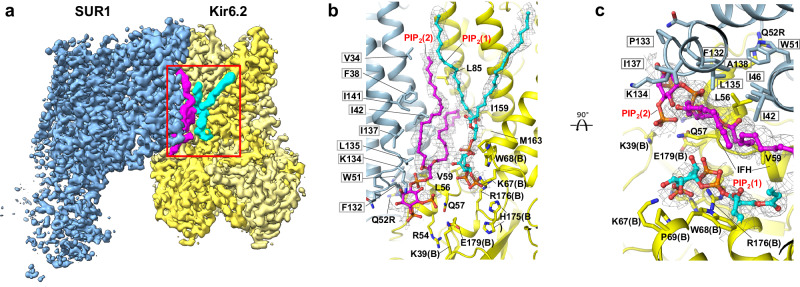
Two PIP_2_ binding sites at the interface of Kir6.2 and SUR1. **a** CryoEM map features of two PIP_2_ molecules colored in cyan and magenta (0.08 V, 4.0σ contour), respectively. **b** Structural model of the PIP_2_ binding pocket (red boxed region in (**a**)) viewed from the side, with cryoEM density of PIP_2_ shown as gray mesh. Residues from both Kir6.2 (adjacent subunit denoted “B”) and SUR1 (gray outline) surrounding bound PIP_2_ molecules are labeled. **c** Structural model of the PIP_2_ binding pocket view from the top (extracellular side).

The cryoEM map of the PIP_2_-bound SUR1/Kir6.2^Q52R^ K_ATP_ channel showed densities of two amphipathic molecules, occupying sites in a large pocket at the Kir6.2-SUR1 interface near the inner leaflet of the membrane bilayer (Fig. 2a). The first amphipathic molecule sits in the predicted canonical PIP_2_ binding site of Kir6.2 based on comparisons with the homologous structures of Kir channels Kir2 and Kir3/GIRK bound to PIP_2_^17–20^ (colored cyan in all relevant figures). The second amphipathic molecule, which has stronger map features (colored magenta in relevant figures) than the one in the canonical PIP_2_ binding site, is nestled between SUR1 and the canonical PIP_2_ binding site and in contact with both Kir6.2 and SUR1 subunits (Fig. 2). We modeled these densities as two C18:0/C20:4 PI(4,5)P_2_ molecules (Figs. 1b, 2b and c, and Supplementary Fig. 3a and b) based on the following. First, C18:0/C20:4 PI(4,5)P_2_ is the dominant species in the brain PIP_2_ present in our sample (~75%)^15^, and both amphipathic molecule densities have map features for the phospho-head groups as well as long acyl chains consistent with C18:0/C20:4 PI(4,5)P_2_ (monomer ID PT5) (Fig. 2b, c, and Supplementary Fig. 3b). Second, the amphipathic molecule densities are less well fit by other common endogenous phospholipids such as phosphatidylethanolamine (PE) or phosphatidylserine (PS) with smaller head groups (Supplementary Fig. 3c, d), or digitonin (Supplementary Fig. 3e), the detergent used to solubilize the membrane. Third, comparison with other open cryoEM K_ATP_ channel structures solved in the absence of long-chain PIP_2_, including the open SUR1/Kir6.2^C166S, G334D^ channel structure and the pre-open SUR1-Kir6.2^H175K^ fusion channel structure, reveals clear differences in the non-protein cryoEM densities in this pocket (Supplementary Fig. 4a, b). However, other residual endogenous lipids or lipid species other than C18:0/C20:4 PI(4,5)P_2_ in the brain PIP_2_ used for the experiment may also contribute to the observed densities. In addition to lipid densities at the SUR1-Kir6.2 interface, three other well-resolved lipid map features at the inner leaflet of the membrane bilayer were bound between SUR1-TMD0 and SUR1-TMD1. These were best modeled by the common phospholipids phosphatidylethanolamine and phosphatidylserine (Supplementary Fig. 3f, g). Of note, lipid or detergent densities were also observed in the outer leaflet space (Supplementary Fig. 3a); however, they were not sufficiently resolved to warrant modeling.

#### Structural analysis surrounding the PIP_2_ head groups

The head group of PIP_2_ at the canonical site forms extensive polar interactions with surrounding residues (Fig. 2b, c). The phosphate groups are coordinated directly by Kir6.2 residues R176 and K67 while Kir6.2-W68 forms close interaction with the inositol ring, although K170, which is about 4.5 Å away and H175, which is about 5.5 Å away, are also in close proximity. Most of the head group-interacting residues are conserved in Kir2 and Kir3 channels with the exception of Kir6.2-P69 and H175, which in Kir2 and Kir3 are an arginine and a lysine, respectively, and shown to coordinate PIP_2_ binding in Kir2 and Kir3^17–20^. The amino acid difference at these two positions would weaken Kir6.2-PIP_2_ interactions, which may explain the lower phosphoinositide head group specificity compared to other Kir channels^33^. Interestingly, Kir6.2-H175 sidechain exhibited two rotameric positions in the PIP_2_-bound SUR1/Kir6.2^Q52R^ channel cryoEM density map: one oriented towards PIP_2_ in the conserved binding site and the other towards E179 in the same Kir6.2 subunit (Fig. 2b). Kir6.2-H175 has been previously implicated in acid-induced activation of K_ATP_ channels between pH 7.4 and 6.8 with a pK of 7.16^39^. Mutating Kir6.2-H175 to lysine, which mimics protonated state of H175, increased basal channel activity and abolished channel activation by pH, suggesting protonation of H175 favors channel opening^39^. Moreover, mutating Kir6.2-E179 to Q greatly attenuated pH-induced channel activation, suggesting Kir6.2-E179 also has a role in acid activation of K_ATP_ channels. Our structural observation that H175 sidechain has interaction with PIP_2_ head group in the canonical site and with Kir6.2-E179 is consistent with the aforementioned mutation-function correlation studies. Since our protein sample was prepared at pH~7.5 (see Methods), which predicts H175 to be largely unprotonated based on the pK of acid activation of the channel of 7.16, it remains to be determined how protonation of H175 at lower pH that activates the channel alters interaction with PIP_2_ and E179 to stabilize channel opening.

The PIP_2_ molecule in the non-canonical site is coordinated by both Kir6.2 and SUR1 subunits (Figs. 1b and 2). Here, the PIP_2_ head group interacts with surrounding residues including K39 and Q57 of Kir6.2, and N131, F132, P133, and K134 of SUR1. Previously we have noted that in closed K_ATP_ structures, SUR1-K134, located in intracellular loop (ICL) 2 of SUR1-TMD0, has its sidechain directed towards cryoEM densities in the predicted PIP_2_ binding pocket tentatively modeled as phosphatidylserines^38^. Based on the observation, we mutated SUR1-K134 to alanine and found it reduced channel *P*_*o*_, which was recovered by adding exogenous PIP_2_^38^. Our PIP_2_-bound SUR1/Kir6.2^Q52R^ open structure now shows that SUR1-K134 is involved in PIP_2_ binding but at the non-canonical site, directly explaining the mutational study result.

#### Structural interactions with the acyl chains of PIP_2_

Most phospholipids present a range of molecular species comprising acyl chains of diverse length and saturation. However, mammalian PIPs show the predominance of a single hydrophobic backbone composed of arachidonoyl (C20:4) and stearoyl (C18:0) chains^15^. In the PIP_2_-bound SUR1/Kir6.2^Q52R^ open K_ATP_ channel structure determined with an enrichment of natural brain PIP_2_ in the membrane, we observed cryoEM densities consistent with branched long acyl chains for both PIP_2_ molecules and modeled them as such to best fit the densities (Fig. 2b and Supplementary Fig. 3b). The PIP_2_ bound at the canonical site mediates interactions between Kir6.2 subunits. While one chain is primarily associated with the outer TM helix of the Kir6.2 subunit that coordinates head group binding (modeled as the arachidonoyl chain), the other chain sits between the TM helicies of the adjacent Kir6.2 subunit (modeled as the stearoyl chain; Fig. 2b). CryoEM density at this canonical PIP_2_ binding pocket was previously reported by our group and others, but in those studies the map features lack lipid acyl tails and could not be ascertained as PIP_2_^30,38,40,41^ (Supplementary Fig. 4a, b). The lipid tails of the PIP_2_ molecule in the non-canonical site are sandwiched between SUR1-TMD0 and Kir6.2-TMs, with one chain running along TM1 and TM4 of SUR1-TMD0 (modeled as the arachidonoyl chain) and the other between TM1 of SUR1-TMD0 and TMs of Kir6.2 (modeled as the stearoyl chain), and have non-polar interactions with SUR1 (V34, F38, I42, P45, I46, L135, I137, A138, I141) and Kir6.2 (L56, V59, I159) respectively (Fig. 2b, c). Long-chain PIP_2_ stably activates K_ATP_ channels^11,12^; in contrast, activation of channels by synthetic short-chain PIP_2_ such as diC8-PIP_2_ is readily reversible^33^. The extensive hydrophobic interactions between channel subunits and the long acyl chains of C18:0/C20:4 PI(4,5)P_2_ observed in our structure may contribute to the apparent increase in stability of channel activation by long-chain PIP_2_.

#### Functional role of the two PIP_2_ binding sites

**Fig. 3:**
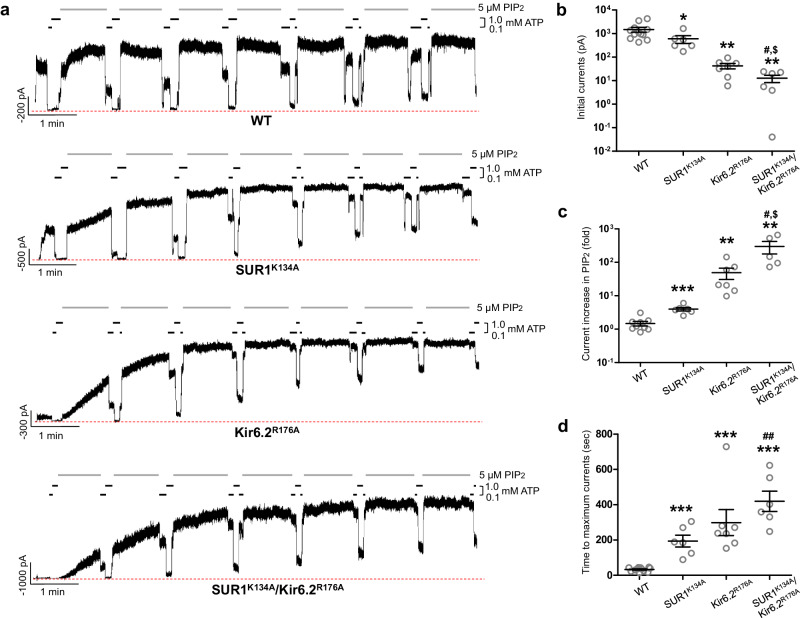
Functional testing of the two PIP_2_ binding sites. **a** Representative inside-out patch-clamp recordings (−50mV, inward currents shown as upward deflections; the red dashed line indicates 0 currents) of various channels: WT, SUR1^K134A^ (the non-canonical PIP_2_ binding site mutation)/Kir6.2, SUR1/Kir6.2^R176A^ (the canonical PIP_2_ binding site mutation), and SUR1^K134A^/Kir6.2^R176A^ double mutant, exposed alternately in 5 μM PIP_2_, 0.1 mM ATP, and 1 mM ATP as indicated by the bars above the recordings. The brief exposures to 0.1 mM and 1 mM ATP between PIP_2_ exposures were used to monitor the gradual decrease in ATP sensitivity as channel opening became increasingly stabilized by PIP_2_. **b** Group data of recordings shown in (**a**) comparing initial currents in K-INT at the time of patch excision. For each group, mean ± SEM of the data points are shown (WT, *n* = 12; SUR1^K134A^, *n* = 6; Kir6.2^R176A^, *n* = 7; SUR1^K134A^/Kir6.2^R176A^, *n* = 6 cells). Statistical significance was tested between mutants and WT or between double and single mutants using two-tailed unpaired *t* test with Welch’s correction, alpha = 0.05. *SUR1^K134A^ vs WT, *p* = 0.0468; ** Kir6.2^R176A^ vs WT, *p* = 0.0016; **SUR1^K134A^/Kir6.2^R176A^ vs WT, *p* = 0.0014; ^#^SUR1^K134A^/Kir6.2^R176A^ vs SUR1^K134A^, *p* = 0.0440; ^$^SUR1^K134A^/Kir6.2^R176A^ vs Kir6.2^R176A^, *p* = 0.0411. Note the *y*-axis in this panel and panel (**c**) is in log scale for better visualization. **c** Comparison of the maximum fold-increase in currents after PIP_2_ exposure in different channels. For each group, mean ± SEM of the data points are shown (WT, *n* = 12; SUR1^K134A^, *n* = 6; Kir6.2^R176A^, *n* = 7; SUR1^K134A^/Kir6.2^R176A^, *n* = 5 cells). Statistical significance was tested between mutants and WT or between double and single mutants using two-tailed unpaired *t* test, alpha = 0.05. ***SUR1^K134A^ vs WT, *p* < 0.0001; ** Kir6.2^R176A^ vs WT, *p* = 0.0029; **SUR1^K134A^/Kir6.2^R176A^ vs WT, *p* = 0.0012; ^#^SUR1^K134A/^Kir6.2^R176A^ vs SUR1^K134A^, *p* = 0.0247; ^$^SUR1^K134A/^Kir6.2^R176A^ vs Kir6.2^R176A^, *p* = 0.0353. **d** Comparison of the time of exposure in PIP_2_ for currents to reach maximum in different channels. For each group, mean ± SEM of the data points are shown (WT, *n* = 12; SUR1^K134A^, *n* = 6; Kir6.2^R176A^, *n* = 7; SUR1^K134A^/Kir6.2^R176A^, *n* = 6 cells). Statistical significance was tested between mutants and WT or between double and single mutants using two-tailed unpaired *t* test, alpha = 0.05. ***SUR1^K134A^ vs WT, *p* < 0.0001; ***Kir6.2^R176A^ vs WT, *p* = 0.0002; ***SUR1^K134A^/Kir6.2^R176A^ vs WT, *p* < 0.0001; ^##^SUR1^K134A^/Kir6.2^R176A^ vs SUR1^K134A^, *p* = 0.0071.

To probe the functional role of the two PIP_2_ binding sites, we compared the PIP_2_ response of WT channels to channels containing the following mutations: Kir6.2^R176A^, which is predicted to weaken the canonical PIP_2_ binding site; SUR1^K134A^, which is expected to weaken the non-canonical PIP_2_ binding site; Kir6.2^R176A^ and SUR1^K134A^, which weaken both PIP_2_ binding sites (Fig. 3a). Exposure to brain-derived C18:0/C20:4 PI(4,5)P_2_ increased activity that is resistant to washout and decreased ATP inhibition over time in WT channels, as has been shown previously^11,12^. Similar effects of PIP_2_ were observed in all three mutant channels (Fig. 3a). However, the initial currents upon membrane excision, the extent of current increase upon PIP_2_ stimulation, and the PIP_2_ exposure time required for currents to reach maximum showed striking differences among the different channels (Fig. 3b–d). WT channels exhibited robust activity upon membrane excision into ATP-free solution, and currents increased by 1.48 ± 0.23-fold (Fig. 3a) that plateaued within 1 min of PIP_2_ exposure. Perturbation of the non-canonical PIP_2_ binding site by SUR1^K134A^ resulted in channels that showed reduced initial currents (596.91 ± 218.23 pA vs. 1488.53 ± 348.86 pA of WT channels), which increased by 3.96 ± 0.47-fold in response to PIP_2_. Perturbation of the canonical PIP_2_ binding site by Kir6.2^R176A^ markedly reduced initial currents (42.44 ± 11.12 pA), which increased by 48.74 ± 18.25-fold after PIP_2_ exposure. Combining SUR1^K134A^ and Kir6.2^R176A^ yielded channels that showed barely detectable currents at patch excision (12.62 ± 4.34 pA) and a dramatic 299.65 ± 121.72-fold current increase by PIP_2_. All three mutants also required longer PIP_2_ exposure to reach maximum currents in the order of double mutant (419.33 ± 57.86 sec) > Kir6.2^R176A^ (298.57 ± 73.76 s) > SUR1^K134A^ (193.83 ± 33.66 s), compared to WT channels (33.17 ± 3.18 sec) (Fig. 3d). These results demonstrate that both PIP_2_ binding sites have functional roles in K_ATP_ channel activity.

### Kir6.2 pore in open conformation

**Fig. 4:**
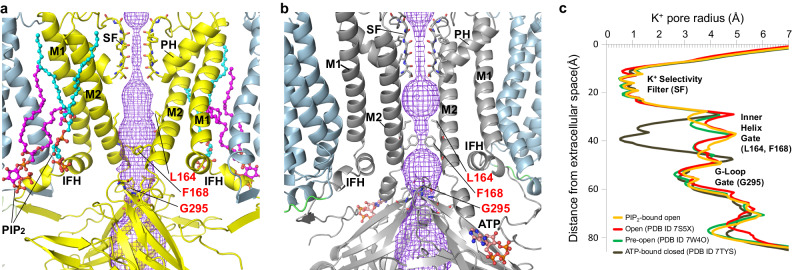
Comparison of the inner helix gate in the open and closed K_ATP_ channel structures. **a** View of the Kir6.2^Q52R^ pore in the PIP_2_-bound SUR1/Kir6.2^Q52R^ open structure. Bound PIP_2_ molecules are shown as cyan and magenta sticks and spheres, and the side chains of Kir6.2^Q52R^ inner helix gating residues L164 and F168 at the helix bundle crossing and G295 at the G-loop are shown as yellow sticks and labeled in red. The pore for the ion pathway (purple mesh) is constricted at the selectivity filter (SF) but open at the inner helix gate (3.3 Å). The Kir6.2^Q52R^ transmembrane α-helices (M1 and M2), the interfacial α-helices (IFH) and the pore α-helices (PH) are labeled. **b** Kir6.2 in an ATP-bound closed state (PDB ID 6BAA) with similar labels as panel a for comparison. **c** Pore radii for ion conduction pathway plotted against the distance from the extracellular opening, with the location of the selectivity filter (residues 130–133), the inner helix gate (residues L164 and F168), and the G-loop gate (residues 294–297) shown. The pore radii were calculated using the program HOLE implemented in Coot and viewed using a MOLE rendering in PyMOL.

**Fig. 5:**
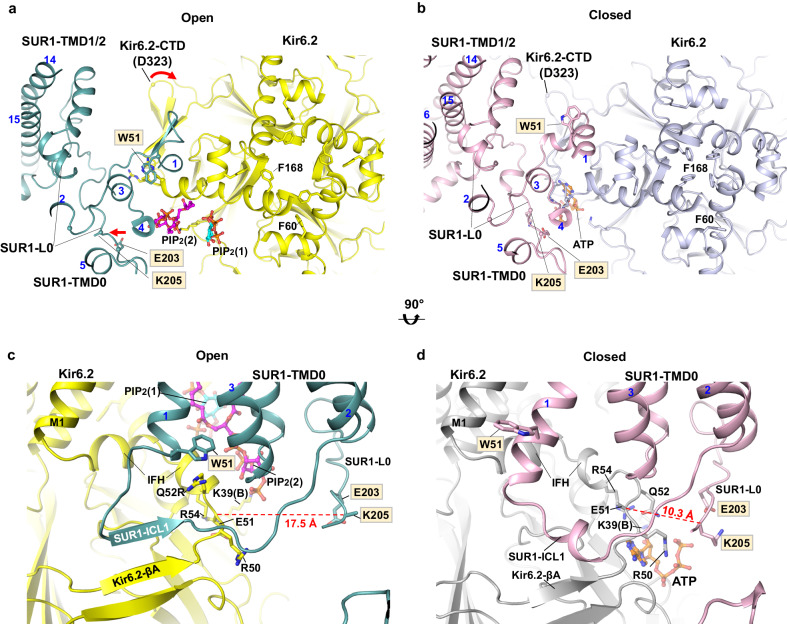
SUR1 and Kir6.2 cytoplasm-plasma membrane interface in open and closed K_ATP_ channel conformations. **a** SUR1-Kir6.2 cytoplasm-plasma membrane interface in open PIP_2_ (cyan and magenta sticks)-bound SUR1/Kir6.2^Q52R^ (SUR1 in teal and Kir6.2 in yellow) structure, and (**b**) closed ATP- (orange carbon sticks) and repaglinide (out of view)-bound WT (SUR1 in pink and Kir6.2 in gray) K_ATP_ channel (PDB ID 7TYS) viewed from the extracellular side. Note reorientation of side chains of the Kir6.2 inner helix gate residue F168 and M1 residue F60, as well as W51 at the bottom of TM1 of SUR1-TMD0 in the two conformations. In (**a**), a 6.4° clockwise rotation of the Kir6.2-CTD comparing the open to the closed conformation is indicated by the red curved arrow (with D323 Cα in each structure marked as spheres), and SUR1-L0, marked by the ATP-binding residue K205 and an adjacent residue E203, movement away from the ATP binding pocket in the open conformation relative to the closed conformation is marked by a red arrow. **c** A side view of the interface in the open conformation. SUR1-intracellular loop 1 (ICL1) forms a β-sheet with Kir6.2-βA just before the interfacial helix (IFH). Remodeling of residues involved in ATP binding and gating compared to the closed conformation in (**d**), including Kir6.2-R50, R54, E51 and K39(B) (“B” denotes the adjacent Kir6.2 subunit) as well as SUR1-E203 and K205 in L0 are shown. Most notably, the Kir6.2 mutant residue Q52R has its side chain reoriented to interact with the side chain of SUR1-W51 which also has its side chain reoriented from the closed conformation. Color scheme same as (**a**). **d** The same side view as in (**b**) but of the closed conformation. In all panels, SUR1 residue labels are outlined in gray. The red dashed line in (**c**) and (**d**) is the distance between the Cα of Kir6.2 E51 and SUR1 K205. In all panels, SUR1 helices are indicated with blue numbers.

In Kir channels, there are three constriction points in the K^+^ conduction pathway: the selectivity filter on the extracellular side, the inner helix gate at the helix bundle crossing where four inner helices (M2) converge, and the G-loop gate in the cytoplasmic pore just below the membrane^13^. The structure of the PIP_2_-bound SUR1/Kir6.2^Q52R^ channel has an open Kir6.2 inner helix gate (Fig. 4a). Clear cryoEM density shows rotation of the Kir6.2-F168 side chains away from the pore’s center, thus causing a dilated pore size (Figs. 4a, 5a; Supplementary Fig. 4c; Supplementary movie 1). The radius of the K^+^ pathway at the inner helix gate is ~3.3 Å, compared to ~0.5 Å in the WT K_ATP_ channel bound to inhibitors we reported previously^38,40^, exemplified by the ATP and repaglinide (RPG) bound structure (PDB ID 7TYS)^38^ (Fig. 4b, c). The radius at the inner helix gate in our current structure is comparable to that in the open structure of SUR1/Kir6.2^C166S,G334D^ (3.3 Å)^28^, and the pre-open structure of SUR1-Kir6.2^H175K^ fusion channel (3.0 Å)^29^ (Fig. 4c). In contrast to the inner helix gate, little difference is observed in the G-loop gate between closed and open structures (Fig. 4), suggesting minimum involvement of the G-loop gate in K_ATP_ channel gating by PIP_2_.

The SUR1/Kir6.2^Q52R^ K_ATP_ sample was purified in the absence of nucleotides; as expected, no ATP cryoEM density was observed at the inhibitory ATP-binding site on Kir6.2^Q52R^. Nonetheless, the open conformation includes structural rearrangements at the ATP binding site that would disfavor ATP binding (Fig. 5a, c and Supplementary Fig. 4c, e). Specifically, the sidechains of Kir6.2-K39 and R50 flip away from ATP-interacting positions, with the K39 sidechain now coordinating PIP_2_ at the non-canonical site; moreover, the sidechain of Kir6.2-E51 forms a salt bridge with R54 to partially occlude the ATP binding pocket (Fig. 5c). Our observation agrees with previous hypotheses that the open channel conformation is not compatible with ATP binding at Kir6.2 and that ATP inhibits the channel by stabilizing the channel in a closed conformation^16^.

Relative to channels bound to inhibitors, in which the Kir6.2-CTD is predominantly positioned close to the plasma membrane (“CTD-up” conformation)^38^, the PIP_2_-bound SUR1/Kir6.2^Q52R^ open channel structure shows the Kir6.2-CTD is also juxtaposing the membrane but further rotated clockwise (viewed from the extracellular side) (compare Fig. 5a and b; Supplementary movie 1). This twisting as well as the other structural changes at the ATP binding site discussed above are similarly observed in the open structure of the SUR1/Kir6.2^C166S,G334D^ channel^28^, and the pre-open structure of the SUR1-Kir6.2^H175K^ fusion channel^29^ (Supplementary Fig. 4d, f). The converging observations in three open structures using different mutant constructs suggest WT K_ATP_ channels undergo similar conformational changes between closed and open states.

### Analysis of SUR1 structure

In the PIP_2_-bound open SUR1/Kir6.2^Q52R^ structure, the two NBDs of SUR1 are not well resolved; in particular, NBD2 is highly dynamic with a local resolution > 5 Å (Fig. 1 and Supplementary Fig. 1d). However, the two NBDs are clearly separated, as predicted since no MgATP or MgADP was added to the sample. Notably, no cryoEM density corresponding to the distal N-terminal peptide of Kir6.2^Q52R^ was observed in the central cleft lined by TM helices from TMD1 and TMD2 of the SUR1-ABC core, even when the map was filtered to 7 Å (Supplementary Fig. 5a); no continuous density corresponding to KNtp was observed at an alternative site to suggest a stable resting position, precluding structural modeling of this flexible domain. The absence of KNtp cryoEM density in the PIP_2_-bound open SUR1/Kir6.2^Q52R^ structure is in contrast to the clear KNtp density observed in the central cleft of SUR1-ABC core in the apo closed SUR1/Kir6.2^Q52R^ structure at comparable resolution and contour level (Supplementary Figs. 2e, 5b). Presence of KNtp in the SUR1-ABC core has been previously reported in inhibitor-bound structures and linked to channel closure^38,41,42^. Worth noting, in a published open K_ATP_ structure where NBDs are bound to MgATP/MgADP and dimerized, KNtp density was also absent^28^. The lack of KNtp in the SUR1-ABC core in the PIP_2_-bound open SUR1/Kir6.2^Q52R^ structure further enforces the correlation between the absence of KNtp in the central cleft of the SUR1-ABC core and channel opening, whether SUR1 NBDs are dimerized or not.

The ABC modules of the four SUR1 subunits in the PIP_2_-bound open SUR1/Kir6.2^Q52R^ map from a C4 non-uniform refinement adopt a propeller-like conformation (Fig. 1a, b). Further 3D classification of the symmetry-expanded particle set revealed three distinct rotational positions of the SUR1-ABC module relative to the Kir6.2/SUR1-TMD0 tetrameric core (Supplementary Fig. 6a, b). Similar conformational dynamics have been reported for the SUR1 NBDs-dimerized, open structure of the SUR1/Kir6.2^C166S,G334D^ channel^28^, thus likely represent intrinsic flexibility of this domain regardless of nucleotide binding and/or NBD dimerization. In addition to the dynamics of the SUR1-ABC module within the PIP_2_-bound open SUR1/Kir6.2^Q52R^ conformation class, more pronounced heterogeneity was also captured in 3D classification of symmetry expanded particles that include all particles from 2D classes (Supplementary Fig. 2). Here, rotation of the SUR1-ABC module is seen correlated with the corkscrew position of the Kir6.2-CTD, with the SUR1-ABC module rotated in towards the Kir6.2 tetramer in the apo closed CTD-down conformation class, and more flung out in the PIP_2_-bound open CTD-up conformation (Supplementary Figs. 2c, d and 6c, d). Such correlated dynamic movements of SUR1 and Kir6.2 may reflect the conformational transition between closed and open channels.

**Fig. 6:**
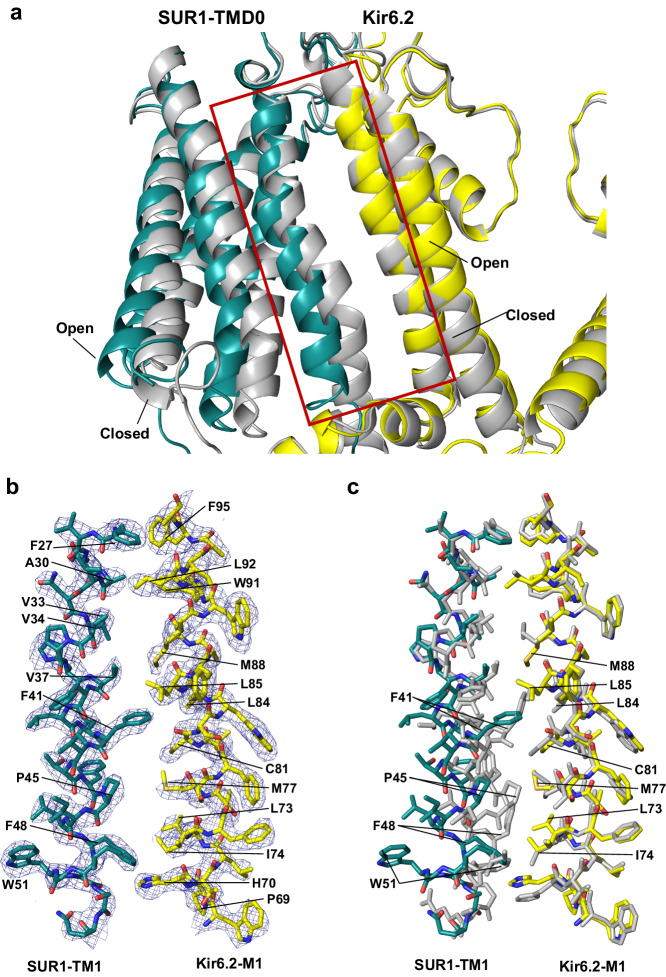
Structural changes between open- and closed-forms of the K_ATP_ channel at the SUR1 and Kir6.2 TM interface. **a** An overlay of the SUR1-Kir6.2 TM interface between the open SUR1/Kir6.2^Q52R^ K_ATP_ channel and the closed K_ATP_ channel (PDB ID 6BAA) showing the outward movement of the cytoplasmic half of the SUR1-TMD0. In the open structure, SUR1 is shown in teal and Kir6.2 in yellow. The closed structure is shown in gray. **b** Close-up view of the open structure in the boxed region in (**a**). CryoEM map (blue mesh, 0.17 V/8.5σ map contour) is superposed with the structural model. For clarity, PIP_2_ is omitted. **c** An overlay of the open with the closed K_ATP_ channel structures in the boxed region in (**a**) (same color scheme) showing significant changes in residues of SUR1-TM1 at the inner leaflet half of the plasma membrane.

Compared to inhibitor-bound closed Kir6.2/SUR1 channel structures (reviewed in^6,7^), SUR1 subunits in the PIP_2_-bound SUR1/Kir6.2^Q52R^ open structure are more tilted away from Kir6.2 and elevated towards the plasma membrane (viewed from the side) such that they are nearly in plane with the Kir6.2 tetramer (compare Fig. 1b and c). Accompanying this upward SUR1 motion, there is an overall rigid-body rotation of SUR1 driven by a rotation of the TMD0 of SUR1 in the open structure relative to closed structures (Fig. 6). At the outer leaflet, SUR1-F41, which interacts with Kir6.2-L84 in the closed conformation, now interacts with Kir6.2-L85 in the open conformation (Fig. 6c). At the inner leaflet, SUR1-TMD0 moves away from Kir6.2 (Fig. 6a, c), creating a space between SUR1-TM1 and Kir6.2-M1. Using PISA analysis^43^, the contact surface area at this interface (between SUR1-TM1 residues 27-54 and Kir6.2-M1 residues 66–96) is 645.6 Å^2^ (ΔG = -20 kcal/mol) in the closed channel structure (PDB ID 6BAA), which is reduced to 395.9 Å^2^ (ΔG = −12 kcal/mol) in the PIP_2_-bound SUR1/Kir6.2^Q52R^ open structure. Binding of a second PIP_2_ in this space, i.e. the non-canonical PIP_2_ binding site (see Fig. 2b), compensates for the lost surface contact between SUR1-TMD0 and Kir6.2, thus stabilizing this interface in the open channel.

Similar structural changes were also described in the pre-open SUR1-Kir6.2^H175K^ fusion structure where SUR1 NBDs are bound to MgATP/MgADP and dimerized^30^ (Supplementary Fig. 4). Dimerization of SUR1 NBDs antagonizes ATP inhibition at Kir6.2 and stimulates K_ATP_ channel activity. It has been proposed that NBD dimerization causes outward bending of the cytoplasmic half of SUR1-TMD0 and the pulling away of K205 in SUR1-L0 from binding ATP at the inhibitory site to stimulate channel activity^30^. Since our PIP_2_-bound open SUR1/Kir6.2^Q52R^ structure does not contain MgATP/MgADP and the NBDs are clearly separated, the similar conformational changes we observed at SUR1-TMD0/L0 are not driven by SUR1 NBD dimerization. One possibility is that MgATP/MgADP and PIP_2_ converge on the same structural mechanism to open the channel, as has been proposed previously based on functional studies^44^.

### Kir6.2-Q52R stabilizes the open conformation by interacting with SUR1-W51

Kir6.2-Q52 is located immediately N-terminal to the interfacial helix (IFH; a.k.a. slide helix) and C-terminal to βA (Fig. 5c, d). In published ATP-bound closed structures, Kir6.2-Q52 lies close to SUR1-E203 in the proximal portion of the SUR1-L0 linker (Fig. 5d). Previous studies have shown that enforcing Kir6.2-Q52 and SUR1-E203 interactions via engineered charged amino acid pair or cysteine crosslinking reduced ATP inhibition IC_50_ by 100-fold or caused spontaneous channel closure in the absence of ATP, respectively^45^. These results indicate that stabilizing the interface between Kir6.2 βA-IFH and SUR1-L0 stabilizes ATP binding and channel closure. In contrast, in the PIP_2_-bound SUR1/Kir6.2^Q52R^ open structure, the SUR1-L0 linker moves ~7 Å towards SUR1 and away from the ATP-binding site on Kir6.2 (compare Fig. 5c and d), with SUR1-E203 and K205 flipped away from Kir6.2 such that SUR1-TMD0 ICL1 (aa 52-60) engages with Kir6.2-βA to form a main chain β-sheet (Fig. 5c). In particular, the sidechain of Kir6.2 residue 52 (Q in WT, R in the mutant) now faces SUR1-TMD0 and interacts with SUR1-W51 (Fig. 5c and Supplementary Fig. 4e), tethering the Kir6.2-CTD to SUR1-TMD0 in a rotated open position.

**Fig. 7:**
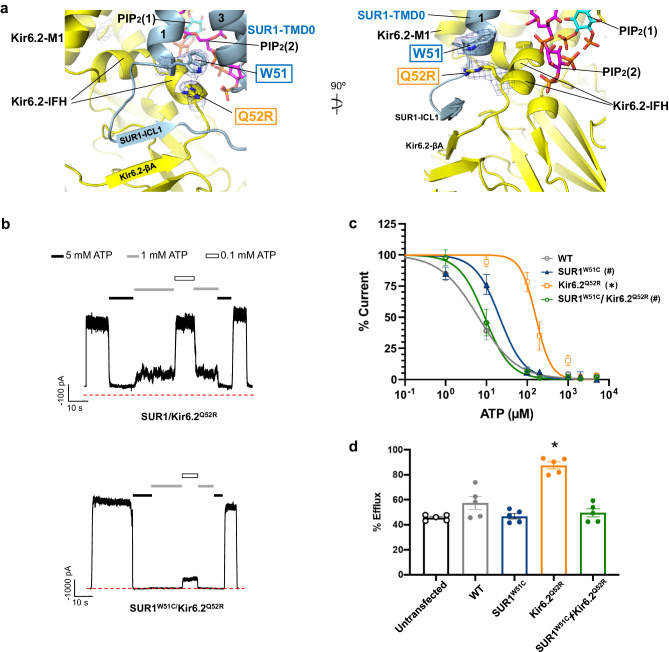
Kir6.2-Q52R interacts with SUR1-W51 to reduce channel sensitivity to ATP inhibition and enhance channel activity. **a** A close-up view of the interaction between Kir6.2-Q52R and SUR1-W51 with cryoEM map (blue mesh, 0.17 V/8.5σ contour) superposed with the structural model in two different angles. **b** Representative inside-out patch-clamp recordings (− 50 mV, inward currents shown as upward deflections; red dashed lines mark baseline 0 currents) of the SUR1/Kir6.2^Q52R^ channel and SUR1^W51C^/Kir6.2^Q52R^ channel exposed to differing concentrations of ATP as indicated by the bars above the recordings. **c** ATP dose response of WT (SUR1/Kir6.2) channels, channels containing the SUR1^W51C^ mutation (SUR1^W51C^), Kir6.2^Q52R^ mutation (Kir6.2^Q52R^), or both SUR1^W51C^ and Kir6.2^Q52R^ mutations (SUR1^W51C^/Kir6.2^Q52R^). Curves were obtained by fitting the data points to the Hill equation (see Methods). Each data point is shown as mean ± SEM (error bar) of WT, *n* = 3, 8, 5, 5, 3, 4, 3 cells for 1 μM, 10 μM, 100 μM, 200 μM, 1 mM, 2 mM, 5 mM, respectively; SUR1^W51C^, *n* = 3, 4, 7, 5, 3, 5, 3 cells for 1 μM, 10 μM, 100 μM, 200 μM, 1 mM, 2 mM, 5 mM, respectively; Kir6.2^Q52R^, *n* = 7, 8, 7, 3, 7, 3 cells for 10 μM, 100 μM, 200 μM, 1 mM, 2 mM, 5 mM, respectively; SUR1^W51C^/Kir6.2^Q52R^, *n* = 3, 7, 5, 3, 9, 3, 4 cells for 1 μM, 10 μM, 100 μM, 200 μM, 1 mM, 2 mM, 5 mM, respectively. Statistical significance in IC_50_ was tested using one-way ANOVA with Tukey’s post hoc test, alpha = 0.05. *p < 0.0001, Kir6.2^Q52R^ vs WT; ^#^*p* < 0.0001, SUR1^W51C^ and SUR1^W51C^/Kir6.2^Q52R^ vs Kir6.2^Q52R^. **d** Rb^+^ efflux of various channels (same labeling as in (**c**)) expressed in COSm6 cells. Untransfected cells were included to show background efflux. Each bar represents means ± SEM of *n* = 5 independent experiments. Statistical significance test using one-way ANOVA shows means are significantly different (*p* < 0.0001, alpha = 0.05). Tukey’s post hoc test shows only SUR1/Kir6.2^Q52R^ is significantly different from WT, *p < 0.0001.

The Kir6.2-Q52R mutation increases *P*_*o*_ and decreases ATP inhibition of K_ATP_ channels in a SUR1-dependent manner^46^. The cation-π interaction observed between Kir6.2-Q52R and SUR1-W51 in the open conformation (Fig. 7a) led us to hypothesize that this interaction may contribute to the gain-of-function gating effect of the Kir6.2-Q52R mutation, by stabilizing the Kir6.2-CTD in a rotated open conformation. To test this, we mutated W51 of SUR1 to cysteine and assessed the ATP sensitivity of channels formed by co-expressing Kir6.2^Q52R^ and SUR1^W51C^ using inside-out patch-clamp recording. SUR1^W51C^ reversed the effect of Kir6.2^Q52R^ such that the ATP sensitivity of the channel resembles WT channels (Fig. 7b). The IC_50_ values of ATP inhibition for WT, SUR1/Kir6.2^Q52R^, SUR1^W51C^/Kir6.2, and SUR1^W51C^/Kir6.2^Q52R^ channels are 6.25 ± 0.44 μM, 161.2 ± 16.23 μM, 20.88 ± 1.96 μM, and 8.98 ± 0.49 μM, respectively (Fig. 7c). Corroborating these findings, in Rb^+^ efflux assays cells co-expressing Kir6.2^Q52R^ and SUR1^W51C^ showed efflux levels similar to cells expressing WT channels, in contrast to the significantly higher efflux in cells co-expressing Kir6.2^Q52R^ and WT-SUR1 (Fig. 7d). These results provide strong evidence that Kir6.2-Q52R interacts with SUR1-W51 to enhance channel activity and reduce ATP inhibition, thus providing an explanation for the SUR1-dependent pathophysiology of this neonatal diabetes mutation.

To test whether the interaction between Kir6.2-Q52R and SUR1-W51 that gives rise to the enhanced activity and reduced ATP sensitivity is dependent on PIP_2_ binding at the two adjacent sites revealed by our structure, we determined the effect of Kir6.2^Q52R^ on the background of SUR1^K134A^, Kir6.2^R176A^, or SUR1^K134A^ and Kir6.2^R176A^, which weaken the non-canonical PIP_2_ binding site, the canonical PIP_2_ binding site, or both. Inside-out patch-clamp recording experiments showed a leftward shift in the dose response of ATP inhibition of the SUR1^K134A^/Kir6.2^Q52R^ channel (IC_50_ = 54.08 ± 7.14 μM, H = 1.55), the SUR1/Kir6.2^Q52R/R176A^ channel (IC_50_ = 17.97 ± 1.20 μM, H = 1.41), and the SUR1K^134A^/Kir6.2^Q52R/R176A^ channel (IC_50_ = 8.30 ± 1.11 μM, H = 1.18) relative to the SUR1/Kir6.2^Q52R^ channel (IC_50_ = 161.20 ± 16.23 μM, H = 2.34) (Supplementary Fig. 7a). SUR1^K134A^ or Kir6.2^R176A^ alone had little effect on the IC_50_ of ATP inhibition (4.12 ± 0.30 μM, H = 1.54, and 11.27 ± 1.95 μM, H = 0.84, respectively, compared to 6.25 ± 0.44 μM, H = 0.93 for WT channels). In agreement, Rb^+^ efflux experiments showed attenuation of the gain-of-function phenotype associated with Kir6.2^Q52R^ when combined with PIP_2_ binding mutations SUR1^K134A^, Kir6.2^R176A^, or both (Supplementary Fig. 7b). Taken together, these results support a role of PIP_2_ at the two binding sites in manifesting the gain-of-function effect of Kir6.2^Q52R^.

## Discussion

In this study, we surmounted the technical difficulties in capturing PIP_2_-bound to K_ATP_ channel, firstly by using a neonatal-diabetes Kir6.2 variant known to enhance channel activity, and secondly by using naturally occurring long-chain PIP_2_ before membrane solubilization, rather than the previously used approach of adding short-chain synthetic diC8-PIP_2_ after channel purification^24,30,47^. The tandem PIP_2_ binding site coordinated by Kir6.2 itself and its regulatory subunit SUR1 uncovers a cooperation between an ion channel and transporter on PIP_2_ binding, and sets apart the K_ATP_ complex in the evolution of K^+^ channels and ABC proteins. The structure offers a resolution to a major problem in the long-standing puzzle of how SUR1, in particular TMD0 of SUR1, modulates the open probability of the Kir6.2 channel^25,26^. Moreover, it suggests a molecular mechanism for how a prominent neonatal diabetes-causing Kir6.2 mutation increases channel activity^46^.

### Distinct PIP_2_ binding pocket in K_ATP_ channels

As a member of the Kir channel family, Kir6.2 was expected to contain a canonical PIP_2_ binding site, akin to that identified in Kir2 and Kir3^17–20^. Our structure confirms that this is indeed the case. Surprisingly, our structure reveals a second, non-canonical PIP_2_ binding site immediately next to the conserved canonical Kir channel PIP_2_ binding site. This non-canonical site is coordinated by both SUR1 and Kir6.2 subunits. Mutational studies support functional importance of PIP_2_ binding at both sites (Fig. 3). In single channel recordings, Kir6.2 channels lacking SUR1 have low *P*_*o*_ with brief openings, in contrast to Kir6.2 channels assembled with SUR1 or SUR1-TMD0, which exhibit ~10-fold higher *P*_*o*_ and long bursts of openings^16,25,26,48^. We propose the low *P*_*o*_ and brief openings in Kir6.2 channels lacking SUR1 result from PIP_2_ binding at the canonical site, which tethers the Kir6.2-CTD near the membrane to rotate to the open position. PIP_2_ binding at this site may not be as strong as in Kir2 and Kir3 channels due to sequence variations at key residues including Kir6.2 P69 and H175 (both positively charged amino acids in Kir2 and 3)^17–20^, explaining the low *P*_*o*_ of Kir6.2. PIP_2_ binding at the non-canonical site formed by SUR1 and Kir6.2 enables SUR1 to stabilize the Kir6.2-CTD in the rotated open position, giving rise to bursts of openings and higher *P*_*o*_.

The space between Kir6.2 and SUR1-TMD0 in the previously published inhibitor-bound closed structure is more constricted than that in the PIP_2_-bound SUR1/Kir6.2^Q52R^ open structure. In a solvent-accessible surface representation of the model for a closed structure (exemplified by the repaglinide/ATP bound PDB ID 7TYS)^38^, PIP_2_ at the non-canonical site would clash with SUR1 (Supplementary Fig. 8b). Inspection of the closed SUR1/Kir6.2^Q52R^ CTD-down conformation (see Supplementary Fig. 2) also finds that PIP_2_ binding at the non-canonical site would clash with SUR1 (Supplementary Fig. 8c), indicating this structure represents an apo state, at least with respect to PIP_2_ binding at the non-canonical site. These observations imply that PIP_2_ moves in and out of the second site as the channel opens and closes. In this regard, it is interesting to note that a conformation-dependent change in the PIP_2_ binding site in the voltage-dependent KCNQ1 (Kv7.1) channel was recently reported, where the movement of the voltage sensing transmembrane helix S4 up or down the membrane is associated with opening or occlusion of the PIP_2_ binding site to open or close the channel, respectively^49^. In our published closed structures without exogenous PIP_2_, there was also lipid density, which we tentatively modeled as two phosphatidylserines^38^. Due to resolution differences, it is not possible to resolve lipid density in the apo closed SUR1/Kir6.2^Q52R^ channel map. Whether other smaller lipids may occupy the space vacated due to incompatibility of PIP_2_ binding at the remodeled non-canonical site in closed channel conformations as channels open and close remains to be determined. Further studies are also needed to determine whether the canonical PIP_2_ binding site is always occupied by PIP_2_ since it appears available in both the open and closed channel conformations (Supplementary Fig. 8).

K_ATP_ channels exhibit lower specificity towards PIPs compared to other Kir channels, and are activated equally well by PI(4,5)P_2_, PI(3,4)P_2_, and PI(3,4,5)P_3_, and also by PI(4)P and long chain (LC)-CoAs^33^. The structural basis underlying the reduced PIPs specificity for K_ATP_ channel activation requires further investigation, but the large size of the binding pocket may accommodate different PIPs and LC-CoAs. Such degeneracy may account for the observation that purified K_ATP_ channels reconstituted in lipid bilayers lacking PIP_2_ exhibited spontaneous single channel openings^28^, in contrast to Kir2 and Kir3 channels which require PIP_2_ for activity^20,50^.

### Mechanism of PIP_2_ and ATP antagonism

PIP_2_ and ATP functionally compete to open or close K_ATP_ channels (Fig. 3a)^11,12^. Kinetic analyses have indicated that PIP_2_ and ATP binding are mutually excluded^16^. Comparison between the PIP_2_-bound SUR1/Kir6.2^Q52R^ open structure and previously published ATP-bound closed structures shows PIP_2_ and ATP antagonism occurs via both binding competition and allosteric mechanisms (Fig. 5 and Supplementary movie 1). At the level of binding competition, both ligands compete for a common binding residue, Kir6.2-K39, which interacts with ATP in the ATP-bound closed structures^38,40^ but with PIP_2_ in the PIP_2_-bound open structure (Figs. 2b and 5c). Previous molecular dynamics simulations, using ATP-bound closed structures of Kir6.2 tetramer^51^ or tetramer of Kir6.2 plus SUR1-TMD0^38^ with a single PIP_2_ molecule docked in the canonical PIP_2_ binding site, found that Kir6.2-K39^51^ or both K39 and R54^38^ switched between ATP binding and PIP_2_ binding. In the PIP_2_-bound SUR1/Kir6.2^Q52R^ open structure, we see Kir6.2-K39, rather than binding PIP_2_ in the canonical site, binds the second PIP_2_ in the non-canonical site. In contrast, Kir6.2-R54 does not appear to be involved in PIP_2_ binding; instead, it interacts with Kir6.2-E51 in the same subunit and E179 in the adjacent Kir6.2 subunit, which stabilizes the interface between the IFH and the C-linker helix (the linker helix connecting M2 and C-terminal cytoplasmic domain of Kir6.2) in the open conformation. Mutation of both K39 and R54 to alanine has been shown to reduce ATP as well as PIP_2_ sensitivities^52^. Our structure clarifies the structural role of K39 and R54 and suggests while K39A likely reduces PIP_2_ sensitivity by weakening PIP_2_ binding at the non-canonical site, R54A may reduce PIP_2_ sensitivity indirectly by disrupting interactions in Kir6.2 that are needed to stabilize the open channel conformation.

Allosterically, PIP_2_ binding diverts ATP binding residues away from the ATP binding pocket to disfavor ATP binding. These include sidechain reorientation of Kir6.2 K39 and R50, and interaction between Kir6.2 R54 and E51 that partially occludes the ATP binding pocket (Fig. 5c). Moreover, in the ATP-bound closed conformation, SUR1-L0 interfaces with Kir6.2-βA, allowing SUR1-K205 to coordinate ATP binding^38,42,45,53^ and stabilize channel closure. However, in the PIP_2_-bound open SUR1/Kir6.2^Q52R^ structure, SUR1-L0 is disengaged from Kir6.2-βA, allowing Kir6.2-CTD to rotate such that ICL1 of SUR1-TMD0 engages with Kir6.2-βA, forming a continuous β-sheet to stabilize the open conformation, which also disfavors ATP binding. Similar changes have been described in the published SUR1/Kir6.2^C166S,G334D^ open structure^28^, and the SUR1-Kir6.2^H175K^ fusion pre-open structure^30^ (Supplementary Fig. 4f). The common structural rearrangements from the closed to the open state are independent of mutations, which suggests the same gating transition occurs in WT channels, and highlights a key role of SUR1 in stabilizing the Kir6.2-CTD in two distinct rotational positions to close or open the K_ATP_ channel.

### Insights on disease mutations

This study sheds light on how the neonatal diabetes-associated Kir6.2^Q52R^ causes gain of K_ATP_ channel function. The strong cation-π interaction engendered by the Kir6.2-Q52R mutation with SUR1-W51 illustrates how changes at this interface have profound effects on channel gating and physiology. In the published SUR1/Kir6.2^G334D,C166S^ open structure and the SUR1-Kir6.2^H175K^ fusion channel pre-open structure Q52 of Kir6.2 is similarly in position to interact with W51 of SUR1^28,29^ (see Supplementary Fig. 4f). The polar-π interaction between glutamine and tryptophan is much weaker than the cation-π interaction between arginine and tryptophan. The difference in binding energy for the relevant gas-phase interactions for polar-π is 1.4 kcal/mol (NH_3_-Benzene), compared to the cation-π interaction energy of 19.0 kcal/mol (NH_4_^+^-Benzene)^54,55^. That Q52 and Q52R in Kir6.2 both interact with W51 of SUR1 suggests an important role of Kir6.2-Q52 in stabilizing the Kir6.2-CTD and SUR1-TMD0 interface for channel opening. Functional studies showing that weakening PIP_2_ binding at either site attenuates both WT and SUR1/Kir6.2^Q52R^ activity are consistent with endogenous PIP_2_ being important for the activity of both WT and mutant channels. In the absence of normal PIP_2_ binding, the gain-of-function effect of Kir6.2^Q52R^ becomes much weaker. The presence of a minor conformation class corresponding to a closed SUR1/Kir6.2^Q52R^ channel with an occluded (apo) non-canonical PIP_2_ binding site (Supplementary Figs. 2, 8c) in our sample further strengthens the notion that Kir6.2^Q52R^ alone is insufficient to support the open channel conformation in the absence of PIP_2_.

**Fig. 8:**
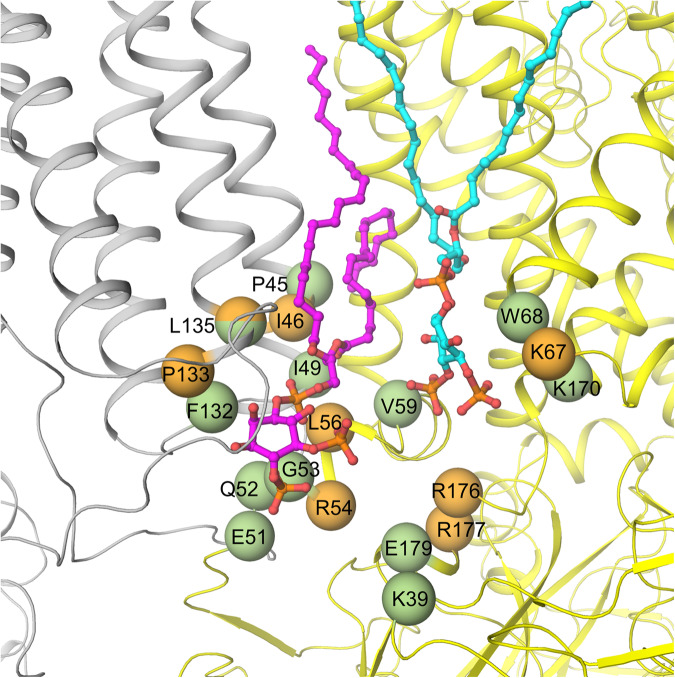
K_ATP_ channel disease mutations near the PIP_2_ binding pocket. Residues with variants that have been reported to cause neonatal diabetes/DEND syndrome are shown as green spheres and congenital hyperinsulinism shown as orange spheres (with the exception of L135, which is colored half green and half orange as mutation at this position has been linked to both diseases depending on the substituting amino acid). Neonatal diabetes/DEND syndrome mutations include SUR1-P45L, I49F, F132L/V, L135P, and Kir6.2-K39R, E51A/G, Q52L/R, G53D/N/R/S/R/V, V59A/G/M, W68C/G/L/R, K170N/R/T, E179A/K. Congenital hyperinsulinism mutations include SUR1-I46T, P133R and L135V, and Kir6.2-R54C, L56G, K67D, R176H, and R177W. SUR1 and Kir6.2 are colored gray and yellow, respectively.

In addition to Kir6.2^Q52R^, many disease mutations are located at the SUR1-Kir6.2 interface seen in the PIP_2_-bound SUR1/Kir6.2^Q52R^ open structure (Fig. 8). These include congenital hyperinsulinism (CHI) associated loss-of-function Kir6.2 mutations: R54C, L56G, K67D, R176H and R177W, and SUR1 mutations: I46T, P133R, and L135V as well as neonatal diabetes (ND) and Developmental delay, Epilepsy, and Neonatal Diabetes (DEND) syndrome associated gain-of-function Kir6.2 mutations: K39R, E51A/G, Q52L/R, G53D/N/R/S/V, V59A/G/M, W68C/G/L/R, K170N/R/T, E179A/K, and SUR1 mutations: P45L, I49F, F132L/V, and L135P (Fig. 8)^56^. Some of these, such as CHI-associated K67D and R176H, and ND/DEND associated K39R, W68C/G/L/R involve residues that coordinate PIP_2_ binding; others, however, likely affect PIP_2_ or ATP gating allosterically by stabilizing channels in closed or open conformations.

In summary, the PIP_2_-bound K_ATP_ channel structure elucidates the intimate partnership between SUR1 and Kir6.2 in coordinating PIP_2_ binding and channel gating. The PIP_2_ binding pocket and the molecular interactions involved in gating identified here provide a framework for designing new K_ATP_ modulators to control channel activity. The finding that SUR1 binds PIP_2_ to regulate Kir6.2 also raises the question of whether PIP_2_ serves structural and functional roles in other ABC proteins, as has been implicated for CFTR^57^.

## Methods

### Cell lines

COSm6 cells (RRID:CVCL_8561) were used for K_ATP_ channel expression. HEK-AD293 cells (RRID:CVCL_0063; from Agilant Technologies catalog number 240085) were used for production of recombinant adenoviruses encoding FLAG-SUR1 and Kir6.2^Q52R^. Both COSm6 and HEK-AD293 cells were cultured in high-glucose DMEM medium (GIBCO) supplemented with 10% Fetal Bovine Serum (Fisher Scientific), 100 U/mL penicillin, and 100 U/mL streptomycin at 37 °C with 5% CO_2_.

### Protein expression and purification

Genes encoding rat Kir6.2^Q52R^ and N-terminal FLAG-tagged (DYKDDDDK) hamster SUR1 were cloned into pShuttle vectors and then the AdEasy vector, and packaged into recombinant adenoviruses in HEK-AD293 cells according to manufacturer’s instructions (https://www.agilent.com/cs/library/usermanuals/public/240009.pdf). Purified recombinant adenoviruses were used to transduce COSm6 cells for protein expression^58^. COSm6 cells grown to mid-log in 15 cm tissue-culture plates were transduced with adenoviruses packaged with Kir6.2, SUR1, and tTA, using multiplicity of infections (MOIs) optimized empirically. Note the pShuttle vector used for SUR1 contains a tetracycline-regulated response element, necessitating co-transduction of a tTA (tetracycline-controlled transactivator) adenovirus for SUR1 expression. At ~24 h post-transduction, cell medium was changed and 100 μM tolbutamide was added to the medium to enhance K_ATP_ channel assembly and expression at the plasma membrane^59^. At ~48 h post-transduction, cells were harvested by scraping and cell pellets were frozen and stored at -80 °C until purification.

For SUR1/Kir6.2^Q52R^ channel purification, cells were resuspended in hypotonic buffer [15 mM KCl, 10 mM HEPES, 0.25 mM dithiothreitol (DTT), pH 7.5] and lysed with a tight-fitting Dounce homogenizer, then centrifuged at 20,000xg for 60 min. The spun-down materials, which include membranes, were re-suspended in buffer containing 0.2 M NaCl, 0.1 M KCl, 0.05 M HEPES pH 7.5, 4% trehalose, and 1 mg/mL brain PIP_2_ (Avanti Polar Lipids, catalog number 840046), and incubated at 4 °C for 30 min before increasing the volume 10x in the same buffer without added lipids but including the detergent digitonin at a final concentration of 0.5%, then membranes were solubilized at 4 °C for 90 min. Solubilized membranes were separated from insoluble materials by centrifugation (100,000xg for 30 min at 4 °C). The soluble fraction was incubated with anti-FLAG M2 affinity agarose for 10 h and eluted at 4 °C for 60 min with a buffer containing 0.2 M NaCl, 0.1 M KCl, 0.05 M HEPES pH 7.5, 0.05% digitonin and 0.25 mg/mL FLAG peptide. Purified channels were eluted at ~170 nM ( ~ 150 μg/ml) and used immediately for cryo-EM grid preparation. K_ATP_ channel particles were also fixed and stained using Uranyl Acetate and sample quality was assessed by negative-stain-EM.

### CryoEM sample preparation and data acquisition

To increase protein adsorption to the cryoEM grids, and also to mitigate selective-orientation of K_ATP_ channel particles that occurs on commercial carbon surfaces, graphene-oxide (GO) grids were prepared as follows^58^. Gold Quantifoil R1.2/1.3 400 mesh grids were cleaned with acetone and glow-discharged for 60 s at 15 mA with a Pelco EasyGlow®, and 4 µL of 1 mg/mL Polyethylenimine (PEI, 40,000 molecular weight) in 25 mM HEPES pH 7.9 was applied to each grid and incubated for 2 min followed by washing with water. Then, 0.1 mg/ml GO was vortexed vigorously and applied to the grid and incubated for 2 min followed by two washes with water. The GO grids were allowed to dry for 15 min and used within 2 h for sample vitrification.

To prepare cryoEM samples, 3 µL of purified K_ATP_ channel complex was loaded onto fresh GO-coated grids for 30 s at 6 °C with a humidity of 100%. Grids were blotted for 2.5 s with a blotting force of -10 and cryo-plunged into liquid ethane cooled by liquid nitrogen using a Vitrobot Mark III (FEI).

Single-particle cryo-EM data was collected on a Titan Krios 300 kV cryo-electron microscope (ThermoFisher Scientific) in the Pacific Northwest CryoEM Center (PNCC), with a multi-shot strategy using beam shift to collect 27 movies per stage shift, assisted by the automated acquisition program SerialEM. Images were recorded on the Gatan K3 Summit direct-electron detector in super-resolution mode, post-GIF (20 eV window), at 105,000x magnification (calibrated image pixel-size of 0.826 Å; super-resolution pixel size 0.413 Å); nominal defocus was varied between −1.0 and −2.5 µm across the dataset. The dose rate was kept around 24 e^-^/Å^2^/s, with a frame rate of 35 frames/sec, and 78 frames in each movie (i.e. 2.2 s exposure time/movie), which gave a total dose of approximately 55 e^-^/Å^2^. Three grids that were prepared in the same session using the same protein preparation were used for data collection, and from these three grids, 2727, 3956 and 1576 movies (8,259 movies total) were recorded.

### CryoEM image processing

Super-resolution dose-fractionated movies were gain-normalized by inverting the gain reference in Y and rotating upside down, corrected for beam induced motion, aligned, and dose-compensated using Patch-Motion Correction in cryoSPARC v4.4.1 (hereinafter referred to as cryoSPARC)^60^ without binning. Parameters for the contrast transfer function (CTF) were estimated from the aligned frame sums using Patch-CTF Estimation in cryoSPARC^60^ and binned by 2 with Fourier cropping. Micrographs were manually curated using sorting by curate exposures. The resulting 5241 dose-weighted motion-corrected summed micrographs were used for subsequent cryo-EM image processing. Particles were picked automatically using template-based picking in cryoSPARC based on 2D classes obtained from cryoSPARC live during data collection. For each of the three sets of data, particles were cleaned by three rounds of 2D classification in cryoSPARC^60^. The combined particle stack contained 70,638 particles, which were then used for ab initio reconstruction in C1 requesting two classes. Only classes that had good alignments and contained full channel particles were included in subsequent rounds of classification. 40,896 particles from the best class were then subjected to ab initio reconstruction in C1 requesting three classes, which gave a class with only side, a class with only top/bottom, and a good class with 23,378 particles. These 23,378 particles were used for a final ab initio reconstruction using three classes, and gave 21,663 particles that sorted into a class with a straight transmembrane region (14,992 particles) and a class where the transmembrane region was more bent with the Kir6.2 core puckered up towards the extracellular space (6,657 particles). Further rounds of ab initio reconstruction resulted in equivalent classes without further improvement in particle classification. The particles were re-extracted using a 600 pixel box at 0.8265 Å/pix, duplicate particles within 20 Å from each other on the micrograph were removed, then used as input for non-uniform refinement^61^ in C1 with the 6,657 particle map reconstruction at 6.4 Å resolution and the 14,115 particle map reconstruction at 3.4 Å resolution. A non-uniform refinement in C4 symmetry imposed with the 14,115 particles resulted in a 2.9 Å resolution reconstructed cryoEM map using cryoSPARC auto mask tightening (Supplementary Fig. 1). The auto-tightened FSC mask excluded the disordered NBD2 (Supplementary Fig. 1c). Masks for FSC calculation that included the full K_ATP_ channel with or without micelle were generated using molmap in UCSF ChimeraX version 1.2 (Chimera)^62^ (Supplementary Fig. 1c), and resampled on the full map grid (600^3^ pixels). The FSC calculated using these static masks yielded 3.3 Å resolution for the full channel without micelle (Supplementary Fig. 1b) and 3.5 Å resolution for the entire particle including the micelle (“Loose” in the FSC plot shown in Supplementary Fig. 1a, b).

To assess conformational heterogeneity for the entire dataset, duplicate and corrupt particles were removed from the set of 70,638 particles from 2D classification to give 66,049 particles and refined using cryoSPARC homogeneous refinement with C4 symmetry imposed (4.0 Å resolution), then 4-fold symmetry expanded to give 264,196 particles (Supplementary Fig. 2a). A mask covering the Kir6.2 tetramer, four TMD0 densities in SUR1, plus a large volume surrounding one of the SUR1 subunits to have a large region covering all possible SUR1 locations, was generated using Chimera and used as a focused map to conduct 3D classification of symmetry expanded particles without particle alignment in cryoSPARC. This yielded two dominant classes: class 1 (106,781 particles), which resembles the open structure derived using the workflow shown in Supplementary Fig. 1a, and class 2 (53,201 particles), which has the Kir6.2^Q52R^ in the CTD-down position resembling previously published apo closed WT channel^38^. Particles from each of these classes were further classified into four classes using cryoSPARC 3D classification, and the classes with the most divergent conformations were compared (Supplementary Fig. 2b-e). The reconstructed map from a C1 local refinement of 12,320 symmetry-expanded particles resembles the published apo closed WT structure (PDB ID 7UQR) and was well-fit by a rigid-body of that structure, but the resolution of the map (6.9 Å) was not sufficient to build a detailed atomic model.

To assess conformational heterogeneity within the high-resolution class of 14,115 particles of full-channel complexes that may have individual SUR1 subunits adopting independent conformations within a single K_ATP_ channel particle, particles were subjected to C4 symmetry expansion and 3D classification without particle alignment in cryoSPARC using the same mask described above. An initial focused 3D classification searching for 5 classes sorted particles into three dominant classes (class 0: 925 particles; class 1: 22474 particles; class 2: 10711 particles; class 3: 25664 particles; class 4: 194 particles). Class 1 to 3 showed three distinct SUR1 conformations, and subsequent local refinement using a loose mask covering the Kir6.2 tetramer, four TMD0 domains of SUR1, and one SUR1, resulted in map reconstructions of 3.12 Å, 3.99 Å, and 3.07 Å resolutions for class 1, 2, and 3, respectively (Supplementary Fig. 6a, b). In all three of these particle classes, the NBDs are separated, but with substantial differences between the relative positions of NBD2, and all have better resolved maps corresponding to the NBD2 than for the overall consensus C4 non-uniform refinement.

In the C4 reconstruction of the full K_ATP_ channel particle, there is good density for nearly every side chain of Kir6.2 except for the N-terminal 31 residues and the C-terminal tail beyond residue 352. TMD0 and the transmembrane helicies of SUR1 also have clear density, with unclear density for the dynamic NBDs and loop regions, especially NBD2 which is poorly resolved due to apparent high flexibility when in the open conformation without NBD dimerization conditions.

To create an initial structural model, PDB ID 6BAA was fit into the reconstructed density for the full K_ATP_ channel particle using Chimera^63^, and then refined in Phenix 1.20.1-4487 (Phenix)^64^ as separate rigid bodies corresponding to TMD (32-171) and CTD 172-352) of Kir6.2 and TMD0/L0 (1-284), TMD1 (285-614), NBD1 (615-928), NBD1-TMD2-linker (992-999), TMD2 (1000-1319) and NBD2 (1320-1582). All sidechains and missing loops that had clear density were then built manually using Coot 0.9.8.1 (Coot)^65^. The resulting model was further refined using Coot and Phenix iteratively until the statistics and fitting were satisfactory (Supplementary Table 1). Two models are presented. The first (PDB ID 8TI2) contains residues 32-352 for Kir6.2, and residues 1-1578 for SUR1 except for an extracellular loop (1043-1060), two loop regions (623-673 and 743-766) in NBD1 and the linker between NBD1 and TMD2 (928-986). Due to the resolution limitation of NBD2, all residues (except for proline) of NBD2 in this model are stubbed at Cβ. The second model (PDB ID 8TI1) has the highly flexible NBD2 removed^66^. The N-terminal peptide of Kir6.2, which in closed NBD-separated structures rests between TMD1 and TMD2 of SUR1^38,41^, is not observed in this conformation.

In addition to protein density, N-acetylglucosamine (NAG), which is the common core of N-linked glycosylation, is modeled in the distinctively large density at the side chain of N10 in each SUR1 (Fig.1b). In addition, densities corresponding to two amphipathic molecules were observed at the interface between Kir6.2 and SUR1 and were modeled as two C18:0/20:4 PI(4,5)P_2_ molecules, which gave the best fit compared to other endogenous phospholipids and the detergent used to solubilize the membrane (see Supplementary Fig. 3a–e). Three other lipid densities per SUR1 monomer were also observed and modeled as phosphatidylserine or phosphatidylethanolamine (Supplementary Fig. 3f, g). The resulting model was further refined using Coot^65^ and Phenix^64,67,68^ iteratively until the statistics and fitting were satisfactory (Supplementary Table 1). All structure figures were produced with Chimera^63^, ChimeraX^62^, and PyMol (http://www.pymol.org). Pore radius calculations were performed with HOLE implemented in Coot^65^.

### Electrophysiology

For electrophysiology experiments, COSm6 cells were co-transfected with various combination of WT or mutant SUR1 and Kir6.2 cDNAs (SUR1 in pECE and Kir6.2 in pCDNA3) along with the cDNA for the Green Fluorescent Protein (to facilitate identification of transfected cells) using FuGENE® 6 (Promega). Mutant SUR1 and Kir6.2 were constructed using QuikChange (mutagenesis primers are shown in Supplementary Table 2). Cells were plated onto glass coverslips 24 h after transfection and recordings made in the following two days. All experiments were performed at room temperature^69^. Micropipettes were pulled from non-heparinized Kimble glass (Fisher Scientific) on a horizontal puller (Sutter Instrument). Electrode resistance was typically 1-2 MΩ when filled with K-INT solution containing 140 mM KCl, 10 mM K-HEPES, 1 mM K-EGTA, 1 mM EDTA, pH 7.3. ATP was added as the potassium salt. Inside-out patches of cells bathed in K-INT were voltage-clamped with an Axopatch 1D amplifier (Axon Instruments). ATP or porcine brain PIP_2_ (Avanti Polar Lipids; prepared in K-INT and bath sonicated in ice water for 30 min before use) were added to K-INT as specified in the figure legend. All currents were measured at a membrane potential of −50 mV (pipette voltage = +50 mV). Data were analyzed using pCLAMP10 software (Axon Instrument). Off-line analysis was performed using Clampfit and GraphPad. Data were presented as mean ± standard error of the mean (S.E.M). ATP inhibition dose-response curves were obtained by fitting data to the Hill equation (I_rel_ = 1/(1 + ([ATP]/IC_50_)^H^)), where I_rel_ is the current relative to the maximum currents in K-INT solution (expressed as % current in Fig. 7c and Supplementary Fig. 7a), IC_50_ is the ATP concentration that causes half-maximal inhibition, and H is the Hill coefficient. Note H was allowed to vary for the curves shown in Fig. 7c and Supplementary Fig. 7a.

### Rb^+^ efflux assay

COSm6 cells were transiently transfected with various combinations of WT or mutant SUR1 and Kir6.2 cDNAs using FuGENE® 6. Untransfected cells were included as background control. Cells were cultured in medium containing 5 mM RbCl overnight. The next day, cells were washed quickly twice in Ringer’s solution (5.4 mM KCl, 150 mM NaCl, 1 mM MgCl_2_, 0.8 mM NaH_2_PO_4_, 2 mM CaCl_2_, 25 mM HEPES, pH 7.2) with no RbCl. Rb^+^ efflux was measured by incubating cells in Ringer’s solution (for experiments shown in Fig. 7d) or Ringer’s solution supplemented with 5 mM glucose (for experiments shown in Supplementary Fig. 7b) over a 40 min period. Inclusion of 5 mM glucose increases intracellular ATP/ADP ratios to suppress channel activity, allowing for better detection of channels with mild gain-of-function phenotype as shown in some of the mutant channels in Supplementary Fig. 7b. At the end of the 40 min incubation, Efflux solution was collected and cells were lysed in Ringer’s solution plus 1% Triton X-100. Rb^+^ concentrations in both the efflux solution and cell lysate were measured using an Atomic Adsorption Instrument Ion Channel Reader (ICR) 8100 from Aurora Biomed. Percent efflux was calculated by dividing Rb^+^ in the efflux solution over total Rb^+^ in the efflux solution and cell lysate. For each experiment, duplicates were included as technical repeats and the average was taken as the experimental value. For all channel combinations, separate transfections were carried out in parallel as biological repeats, as specified in the figure legends. Data were presented as mean ± standard error of the mean (S.E.M) and statistical analysis was performed by one-way ANOVA with Tukey’s post hoc test in GraphPad.

### Reporting summary

Further information on research design is available in the Nature Portfolio Reporting Summary linked to this article.

### Supplementary information

Supplementary Information

Peer Review File

Description of Additional Supplementary Files

Movie 1

Reporting Summary

### Source data

Source Data

## Data Availability

The data that support this study are available from the corresponding authors upon request. The cryo-EM maps have been deposited in the Electron Microscopy Data Bank (EMDB) as EMD-41278 (NBD2 modeled as main chain atoms only), EMD-41277 (NBD2 not modeled), and EMD-43766 (apo closed SUR1/Kir6.2Q52R). Structural models have been deposited in the Protein Data Bank (PDB) under accession code 8TI2 (NBD2 modeled as main chain atoms only), and 8TI1 (NBD2 not modeled). Previously published structures referred to in this article include: 7UQR and EMD-26320; 7TYS and EMD-26193 ; 6BAA and EMD-7073; 7W4O and EMD-32310; 7S5X and EMD-24842. The source data underlying Figs. 3b–d, 7c and d, and Supplementary Fig. 7 are provided as a Source Data file. Source data are provided with this paper.
