# Supplementary material for: Structure of an open KATP channel reveals tandem PIP2 binding sites mediating the Kir6.2 and SUR1 regulatory interface
Source: Nat Commun. 2024 Mar 20;15:2502. doi: 10.1038/s41467-024-46751-5 (PMC10954709; doi:10.1038/s41467-024-46751-5)
Supplement: Supplementary file 1 — Supplementary Information [file 41467_2024_46751_MOESM1_ESM.pdf]

Supplementary Information

Supplementary Figure 1

Supplementary Figure 2

Supplementary Figure 3

Supplementary Figure 4

Supplementary Figure 5

Supplementary Figure 6

Supplementary Figure 7

Supplementary Figure 8

Supplementary Table 1

Supplementary Table 2

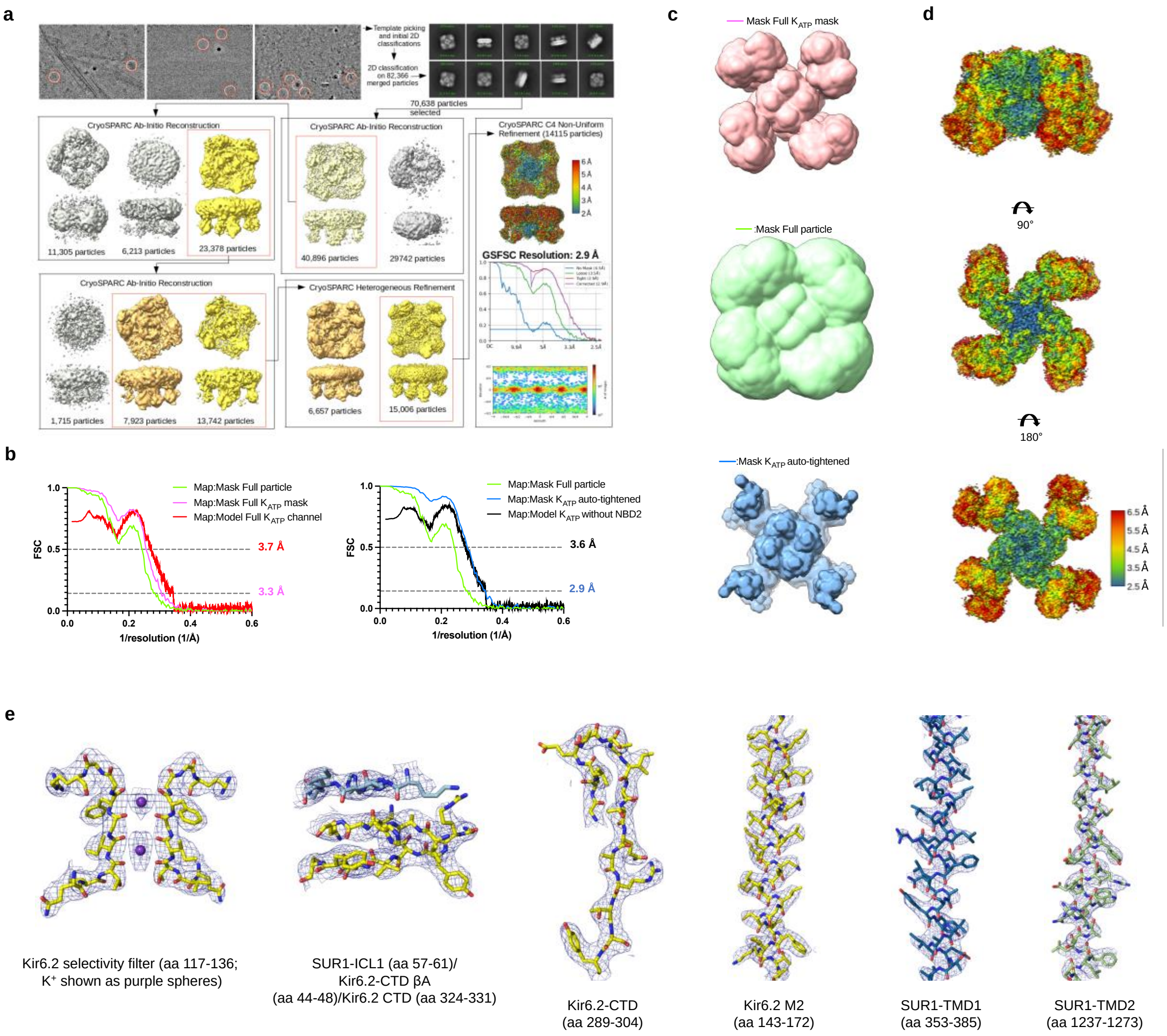

Supplementary Figure 1. CryoEM data processing. (a) Workflow showing the total number of particles yielding the 2.9 Å C4 Non-uniform refinement reconstruction is 14,115 (FSC auto mask for GSFSC calculation). The three raw images on the top left corner serve to illustrate the particles on GO layer (GO edges are seen in the left micrograph) selected (red circles) for processing **(b)** Fourier Shell Correlation (FSC) curves between two independent half-maps calculated within masks shown in (c), and for model:map (unmasked) for the full K_{ATP} channel model and map (PDB ID 8TI2; EMD-41278) (*left*), and for the K_{ATP} channel without NBD2 (PDB ID 8TI1) (*right*). **(c)** Masks used for FSC calculations: full particle including micelle (green), a mask for full K_{ATP} channel without the micelle (pink), and a mask created by cryoSPARC auto-mask tightening that excludes NBD2 (blue), shown in cytoplasmic view. **(d)** The local resolution estimate for the reconstructed map at 4 σ (0.08 V) contour, with micelle density not shown, in side view (*left*), top view (*middle*) and bottom view (*right*). No local filtering or local sharpening was used for visualization. Local resolution estimates were calculated in cryoSPARC and visualized in ChimeraX. FSC curves were calculated in Phenix and cryoSPARC. **(e)** Representative close-up views of cryoEM density map to model fit. All maps are contoured to 8.5 σ, 0.17 V.

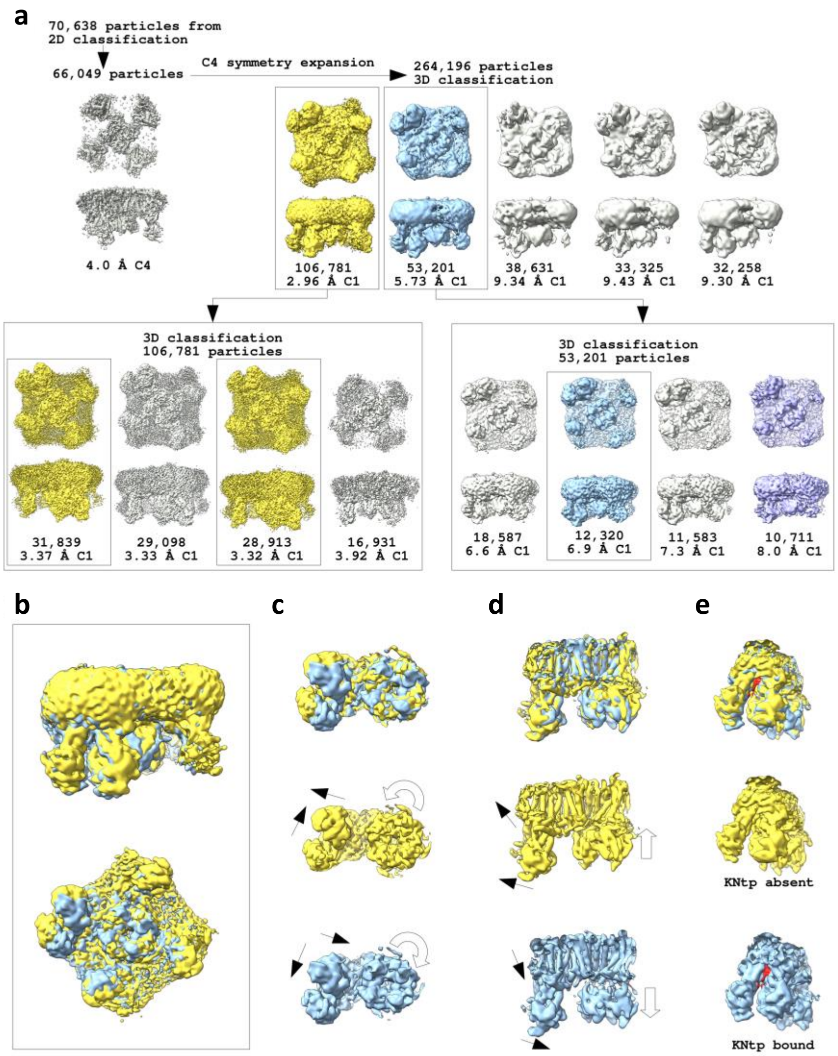

Supplementary Figure 2. Distinct conformation classes revealed by symmetry expansion and focused 3D classification. (a) Workflow to obtain distinct conformation classes (details described in Methods). **(b)** Overlay of full channel map generated by combining the two yellow classes from the second round of 3D classification (shown in yellow) and the blue map from the second round of 3D classification (shown in blue) viewed from the side and the bottom. The yellow map resembles the PIP_2-bound open structure derived from C4 non-uniform refinement (Fig.1a, Supplementary Fig.1a), while the blue map resembles previously published closed apo WT structure (PDB ID 7UQR), and will be referred to as apo SUR1/Kir6.2^{Q52R} structure. **(c)** Cytoplasmic view of the Kir6.2^{Q52R} tetramer plus one SUR1 in the two maps superimposed (top) or separately. In the blue map, Kir6.2^{Q52R}-CTD is rotated clockwise relative to the yellow map. Also in the blue map, the NBDs of SUR1 are closer to the Kir6.2^{Q52R} tetramer and SUR1 is rotated opposite of Kir6.2^{Q52R}-CTD, compare to the yellow map. **(d)** Side view of the maps in (c) showing Kir6.2^{Q52R}-CTD is docked up to the membrane in the yellow map, and extended down from the plasma membrane in the blue map. Also, SUR1 is tilted away from the Kir6.2^{Q52R} tetramer in the yellow map, but tilted towards the Kir6.2^{Q52R} tetramer in the blue map. The curved and straight open arrows mark the relative rotation and translation from the membrane of the Kir6.2^{Q52R}-CTD. The solid arrows indicate relative movements of the SUR1. **(e)** Side view of the SUR1 ABC core showing the presence of cryoEM density corresponding to the Kir6.2^{Q52R} N-terminal peptide (KNtp, red density) in the apo closed SUR1/Kir6.2^{Q52R} map (blue) but absence of KNtp density in the open SUR1/Kir6.2^{Q52R} map (yellow).

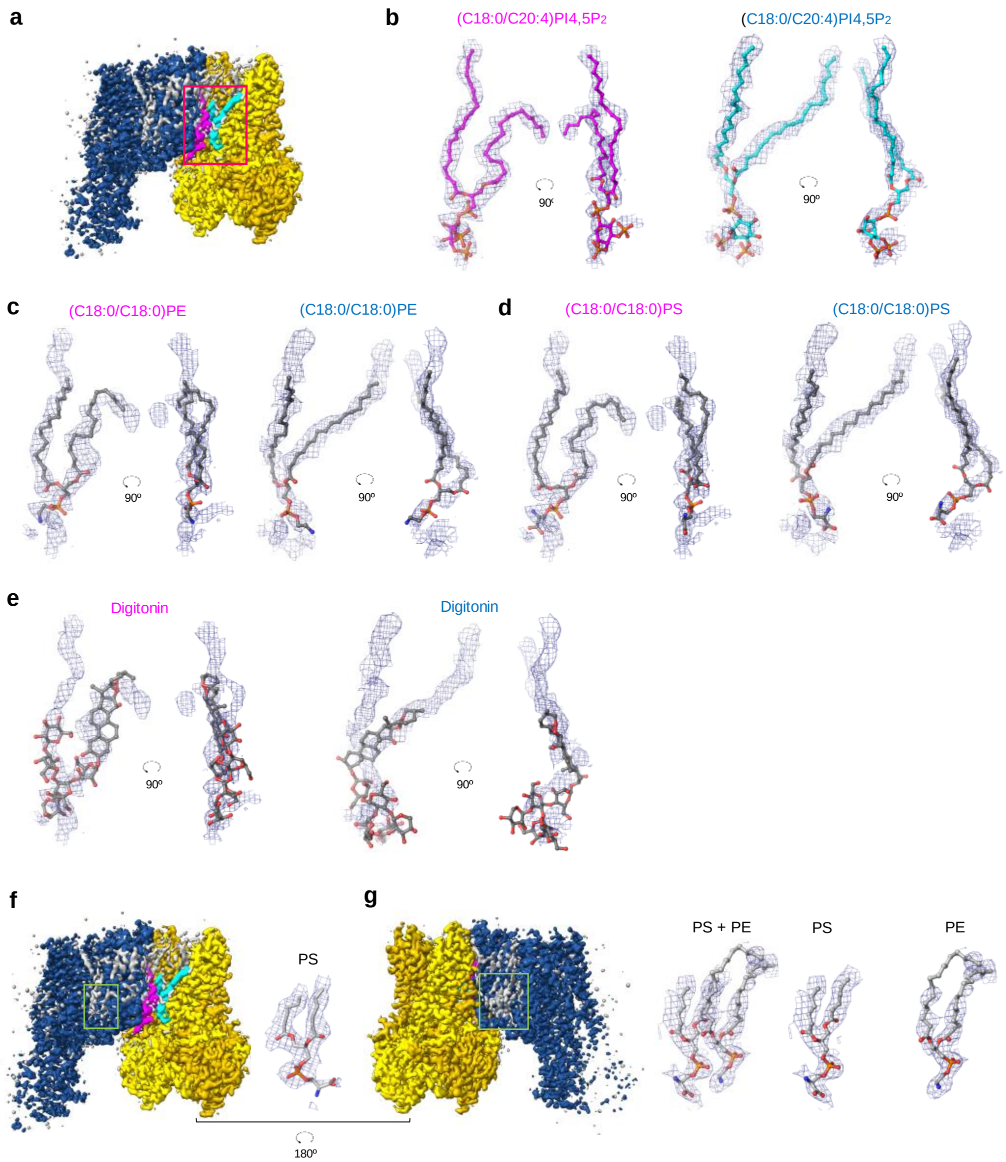

Supplementary Figure 3. CryoEM lipid density fitting. (a) CryoEM map reconstructed from symmetry expansion of the ~14k particles followed by local refinement with a focused mask of Kir6.2 tetramer plus one SUR1, with density modification in Phenix and map segmentation in Chimera, shows the cryoEM densities of SUR1 and the Kir6.2 tetramer colored blue and yellow, respectively. Densities corresponding to two amphipathic molecules at the Kir6.2-SUR1 interface are shown in magenta and cyan, and other likely lipid densities are colored grey (0.08V/4σ contour). **(b-e)** Fitting of the magenta and cyan densities boxed in red in (a) to C18:0/C20:4 PI(4,5)P_2 (b), C18:0/C18:0 PE (c), C18:0/C18:0 PS (d), or digitonin (e) in two different rotational views. Note the C18:0 acyl chains of PE and PS are chosen based on documented common acyl chains of these lipids found in mammalian cell membranes and fit to the cryoEM densities. **(f)** Isolated lipid density located in the inner leaflet between SUR1-TMD0 and SUR1-TMD1 fit with a model of phosphatidylserine (PS, grey carbons, 0.08V/4σ contour). **(g)** Rotation of the map 180° relative to panel (f) reveals two adjacent lipid densities (grey, 0.08V/4.0σ contour) primarily associated with the TMD0 domain, which are modeled as phosphatidylserine (PS) and phosphatidylethanolamine (PE). In (f) and (g), the acyl chains are only modeled up to where cryoEM densities are clear and are shorter than those used for modeling the magenta and cyan densities in (a). Note lipid or detergent densities are also observed in the outer leaflet space; however, they are not sufficiently resolved for modeling.

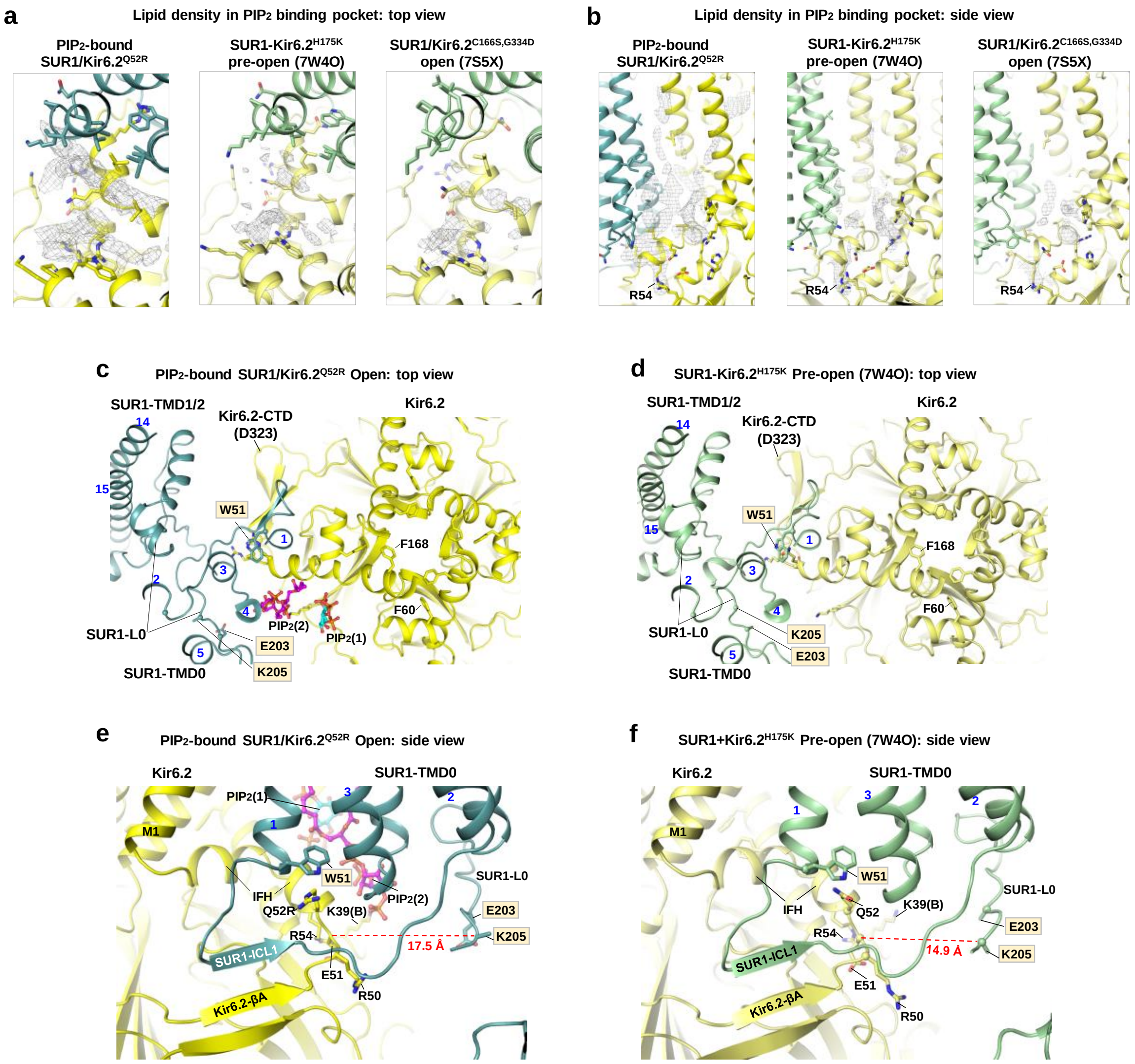

Supplementary Figure 4. Comparison of PIP_2-bound open K_{ATP} channel structure with other open K_{ATP} structures. (a, b) Comparison of the lipid density (grey mesh) in the PIP_2 binding pocket in the PIP_2-bound SUR1/Kir6.2^{Q52R} structure, SUR1-Kir6.2^{H175K} fusion channel pre-open structure (in the presence of diC8-PIP_2; EMD-32310, PDB ID 7W4O), and SUR1/Kir6.2^{C166S,G334D} open structure (no PIP_2 added; EMD-24842, PDB ID 7S5X), viewed from the top (a) and the side (b). In (b), the cryoEM density for Kir6.2 R54 is shown as a reference. **(c, d)** Top view of PIP_2-bound SUR1/Kir6.2^{Q52R} open channel (c) and SUR1-Kir6.2^{H175K} fusion pre-open channel (PDB ID 7W4O) (d), showing similarities in side chain orientations for Kir6.2 F60 and F168 (the gate residue at the helix bundle crossing) and SUR1-W51, and rotation position of the Kir6.2 cytoplasmic domain (CTD) marked by residue D323. **(e, f)** Comparison of the two structures in (c) and (d) viewed on the side, showing close proximity of SUR1-W51 to Kir6.2-Q52R (e) or Q52 (f), as well as the widening of the ATP binding pocket marked by the distance (dashed red line) between the Cα atoms of Kir6.2-E51 and SUR1-K205.

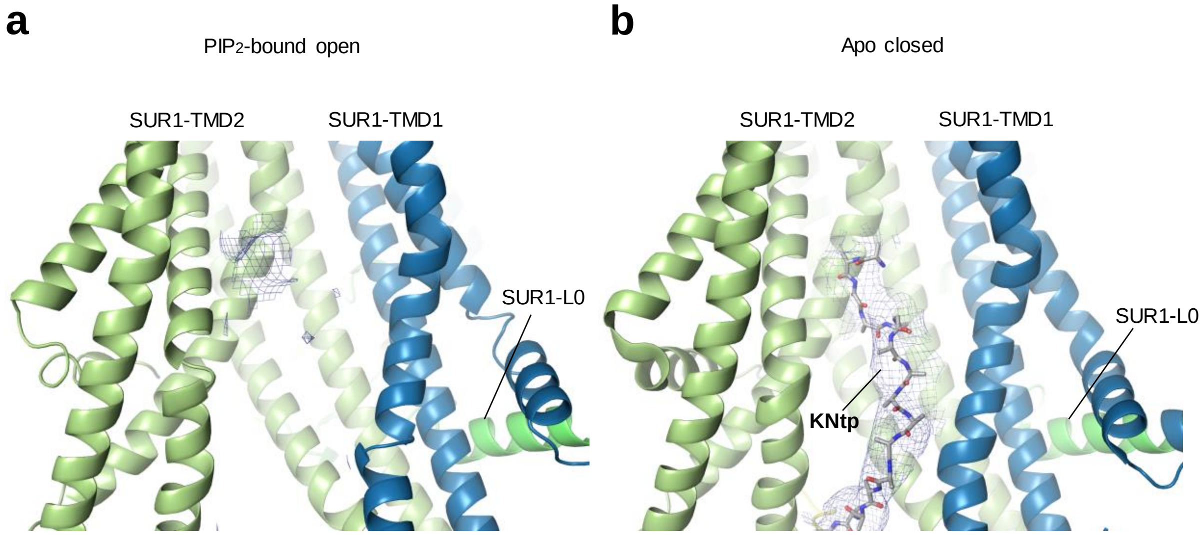

Supplementary Figure 5. Comparison of KNtp density in the SUR1-ABC core in the PIP_2-bound open and the apo closed SUR1/Kir6.2^{Q52R} structures. (a) In the PIP_2-bound SUR1/Kir6.2^{Q52R} open channel (the yellow map in Supplementary Fig.2b), no KNtp density is observed, consistent with the absence of the Kir6.2^{Q52R}-Ntp or a highly flexible Kir6.2^{Q52R}-Ntp. Note the density protruding from SUR1-TMD2 TM helix corresponds to W1297 with some R1145 contribution. **(b)** In the apo SUR1/Kir6.2^{Q52R} closed channel (the blue map in Supplementary Fig.2b), a peptide density corresponding to Kir6.2^{Q52R}-Ntp is clearly present. For density shown in the KNtp binding cleft in SUR1, both maps were filtered to 7 Å and contoured to 6.5 σ.

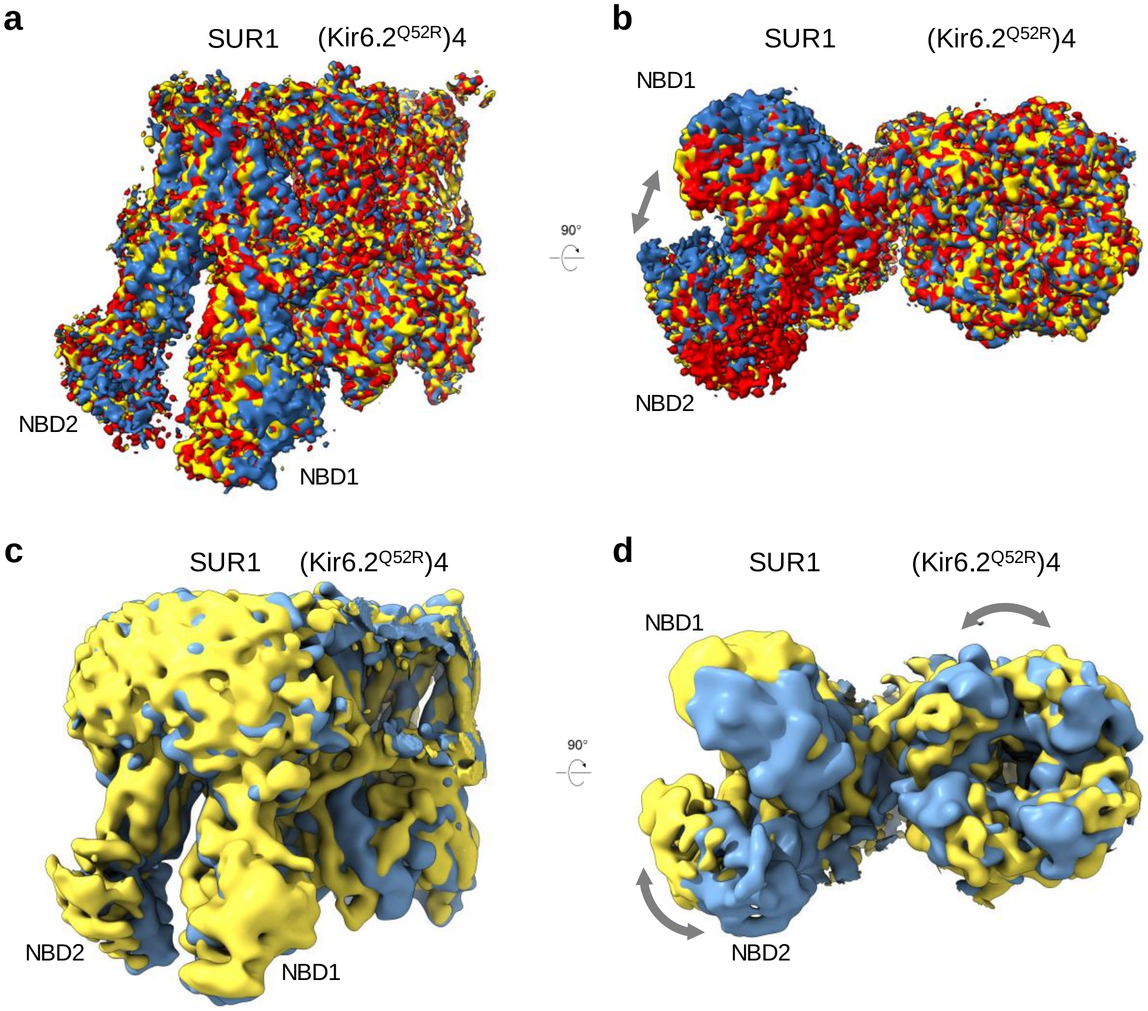

Supplementary Figure 6. Conformational dynamics of the SUR1 subunit revealed by CryoSPARC 3D classification. (a,b) Dynamic range of SUR1 within the final open particle class (Supplementary Fig. 1) was determined by symmetry expansion followed by 3D classification. The 14,115 particles within the final open class gave 56,460 particles, which were subjected to 3D classification with a focused mask of Kir6.2 tetramer plus one SUR1. Three unique NBD-separated SUR1 positions were resolved into three classes. Reconstructed maps of class 1 (3.0 Å resolution, 23,454 particles, red map), class 2 (3.3 Å resolution, 16,863 particles, yellow map) and class 3 (3.4 Å resolution, 13,604 particles, blue map) reconstructed from a local non-uniform refinement with density modification in Phenix. (a) Transmembrane view shows the variability in SUR1 including the transmembrane regions of TMD1 and TMD2, in an open Kir6.2 tetramer position. (b) View from the cytoplasmic side highlights the difference in the SUR1-NBD positions within the open particle class, with mobility seen in both SUR1-NBD1 and SUR1-NBD2. The grey arrow indicates the direction of the NBD position differences. **(c,d)** The reconstructed maps for the PIP_2-bound open class (yellow, the same as the yellow map in Supplementary Fig. 2b, 7.0 Å filtered resolution) and apo closed class (blue, the same as the blue map in Supplementary Fig. 2b, 7.0 Å filtered resolution) within the SUR1/Kir6.2^{Q52R} dataset (workflow in Supplementary Fig. 2), show the relative SUR1 and Kir6.2 domain positions as the channel transitions between open and closed conformations. (c) Transmembrane view shows the difference in the SUR1-NBD position and the rotation of the Kir6.2-CTD. (d) View from the cytoplasmic side highlights the difference in the SUR1-NBD positions relative to the Kir6.2 subunit, with the open conformation having the SUR1-NBD further away from the Kir6.2 cytoplasmic domain, which is rotated counterclockwise relative to the closed position. The grey curved arrows indicate the rotational position difference in Kir6.2^{Q52R}-CTD and SUR1-NBDs.

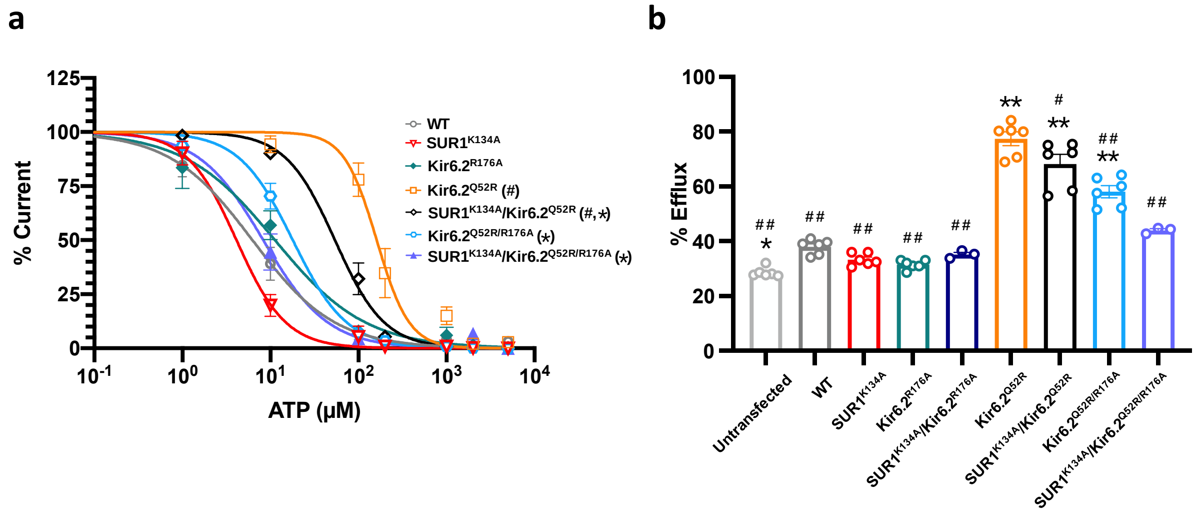

Supplementary Figure 7. Perturbation of PIP$_2$ binding residues diminished the gain-of-function effect of Kir6.2^{Q52R}. (a) ATP dose response of WT (SUR1/Kir6.2) channels, and channels containing the PIP$_2$ binding site mutations SUR1^{K134A} (SUR1^{K134A}) or Kir6.2^{R176A}, as well as the gain-of-function disease mutation Kir6.2^{Q52R} alone or in combination with single PIP$_2$ binding site mutations (SUR1^{K134A}/Kir6.2^{Q52R}, or Kir6.2$^{Q52R/R176A}$) or double PIP$_2$ binding site mutations (SUR1^{K134A}/Kir6.2$^{Q52R/R176A}$). Curves were obtained by fitting the data points to the Hill equation (see Methods). Each data point is shown as mean ± SEM (error bar) of the number of cells (n = 3-8) shown in the Data Source File, with the exception that data points for 1 µM and 1 mM of SUR1^{K134A}/Kir6.2$^{Q52R/R176A}$ only have one cell. Statistical significance in IC$_{50}$ was tested using one-way ANOVA with Tukey's post hoc test, alpha = 0.05. *p < 0.0001, Kir6.2^{Q52R} vs WT; #p < 0.0001, SUR1^{W51C} and SUR1^{W51C}/Kir6.2^{Q52R} vs Kir6.2^{Q52R}. **(b)** Rb$^+$ efflux of various channels [same labeling as in (a)] expressed in COSm6 cells measured in Ringer's solution supplemented with 5 mM glucose. Untransfected cells were included to show background efflux. Each bar represents means ± SEM of n = 6 (untransfected, WT, SUR1^{K134A}, Kir6.2^{R176A}, Kir6.2^{Q52R}, SUR1^{K134A}/Kir6.2^{Q52R}, Kir6.2$^{Q52R/R176A}$) or n = 3 (SUR1^{K134A}/Kir6.2^{R176A}, SUR1^{K134A}/Kir6.2$^{Q52R/R176A}$) independent experiments. Statistical significance test using one-way ANOVA shows means are significantly different (p < 0.0001, alpha = 0.05). Tukey's post hoc test is used for pairwise comparison. *p = 0.0216, untransfected vs WT; **p < 0.0001 mutant vs WT; $^{\#}p$ = 0.0266, SUR1^{K134A}/Kir6.2^{Q52R} vs Kir6.2^{Q52R}; $^{\#\#}p$ < 0.0001, all other groups vs Kir6.2^{Q52R}.

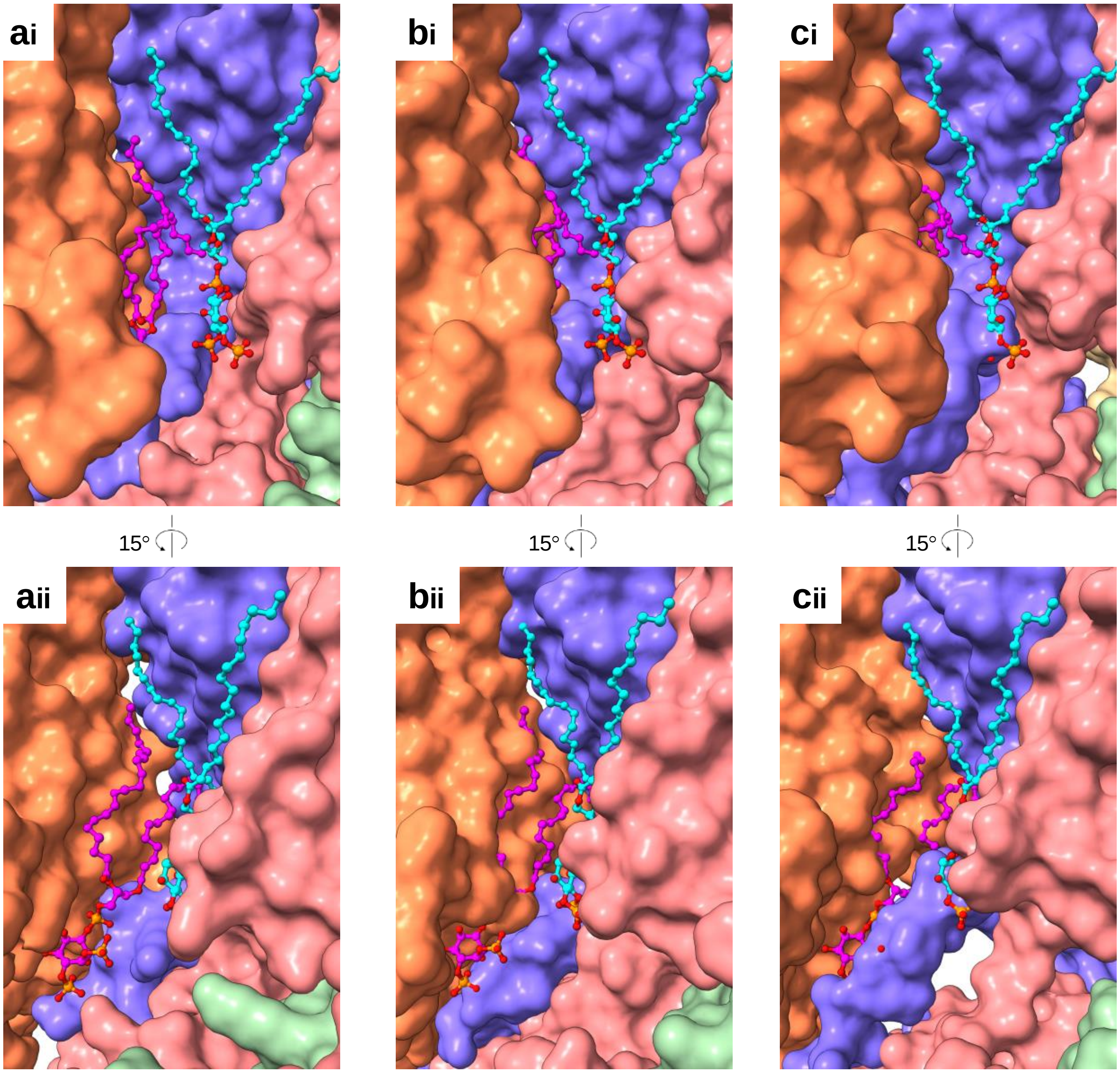

Supplementary Figure 8. The K_{ATP} channel closed conformations clash with the novel second PIP_2 molecule, but both the open and closed conformations could accommodate the conserved first PIP_2. (a) The open SUR1/Kir6.2^{Q52R} K_{ATP} channel with the conserved first PIP_2 (cyan carbons) and novel second PIP_2 (magenta carbons) shown interacting with Kir6.2 subunits (pink, green and blue surface) and the SUR1 subunit (orange surface), with the two panels rotated ~15° to show the surface of each PIP_2 site. **(b)** The closed K_{ATP} channel bound to repaglinide and ATP (PDB ID 7TYS, with Kir6.2-CTD in the up position) aligned with the PIP_2 molecules of the open channel shows that the closed K_{ATP} channel accommodates the conserved first PIP_2 but clashes with the novel second PIP_2. **(c)** The apo closed SUR1/Kir6.2^{Q52R} K_{ATP} channel (with Kir6.2-CTD in the down position, similar to previously reported apo WT structure PDB ID 7UQR; see Fig.S2) also shows clash with PIP_2 at the second site but not the first site.

Supplementary Table 1. Model statistics of the PIP_2-bound SUR1/Kir6.2^{Q52R} structure

**Data Collection**			
Microscope	Titan Krios		
Voltage (kV)	300		
Camera	Gatan K3		
Camera mode	Super-resolution		
Defocus range (µm)	-1.0 ~ -2.5		
Movies	5241		
Frames/movie	78		
Exposure time (s)	2.2		
Frame rate (/s)	35		
Magnified pixel size (Å)[a]	0.826		
Total dose (e^-/Å^2)	~55		
**Reconstruction**			
Software	Cryosparc 4.4.1	Cryosparc 4.4.1	Cryosparc 4.4.1
Symmetry	C4	C4	C1 (C4 expanded particles)
Mask	Full K_{ATP}	autoFSC tightened	$(Kir6.2)_4$+SUR1
Particles refined	14,115	14,115	12,320
EMDataResource	EMD-41278	EMD-41277	EMD-43776[b]
Resolution (masked)	3.3 Å	2.9 Å	6.9 Å
**Refinement Statistics**			
Software	Phenix 1.20.1-4487	Phenix 1.20.1-4487	
PDB ID[c]	8TI2	8TI1	
Atoms	50210	45034	
Protein residues	6896	5848	
Map CC (masked)	0.79	0.81	
FSC model (0.143, 0.5)	3.0, 3.7 Å	2.9, 3.6 Å	
Clash score	6.33	6.72	
Molprobity score	2.04	2.08	
Cβ deviations	0	0	
Rotamer outliers (%)	3.22	3.22	
ADP (mean protein)	172.4	164.4	
ADP (mean ligands)	154.9	154.9	
**Ramachandran**			
Outliers	0.13	0.17	
Allowed	4.23	4.44	
Favored	95.64	95.39	
**Bonds (RMSD)**			
Length (Å)	0.002	0.003	
Bond angles	0.463	0.477	

[a]Super-resolution pixel size 0.4130 Å
[b]EMD-43776 is the apo SUR1/Kir6.2^{Q52R} in the Kir6.2-CTD down closed conformation; the resolution is too low for modeling.
[c]PDB ID 8TI2/EMD-41278 contains the full channel and full K_{ATP} mask; PDB ID 8TI1/EMD-41277 has the flexible NBD2 (residue 1317 to C-terminus) removed and the auto-FSC tightened mask.

Supplementary Table 2. Oligonucleotides used for site-directed mutagenesis

Mutation	Forward	Reverse
Kir6.2-Q52R	CAAGAACATCCGAGAGCGGGGC CGCTTCCTGCAAG	CTTGCAGGAAGCGGCCCCGCTCTC GGATGTTCTTG
Kir6.2-R176A	ACAGGCCCATGCGCGGGCAGAA	TTCTGCCCGCGCATGGGCCTGT
SUR1-W51C	CCTCTTCATCGGATGCGGCAGCC AGAG	CTCTGGCTGCCGCATCCGATGAAG AGG
SUR1-K134A	GACCTCCAACTTCCCCGCGCTTT TGATCGCTCTG	CAGAGCGATCAAAAGCGCGGGGAA GTTGGAGGTC
