# Supplementary material for: Structure of an open KATP channel reveals tandem PIP2 binding sites mediating the Kir6.2 and SUR1 regulatory interface
Source: Nat Commun. 2024 Mar 20;15:2502. doi: 10.1038/s41467-024-46751-5 (PMC10954709; doi:10.1038/s41467-024-46751-5)
Supplement: Supplementary file 3 — Description of Additional Supplementary Files [file 41467_2024_46751_MOESM3_ESM.pdf]

Description of additional supplementary material

File Name: Movie 1

Description: Morph between PIP2-bound open SUR1/Kir6.2Q52R channel structure and ATP/Repaglinide-bound closed WT channel structure.
