# Supplementary material for: Structure of an open KATP channel reveals tandem PIP2 binding sites mediating the Kir6.2 and SUR1 regulatory interface
Source: Nat Commun. 2024 Mar 20;15:2502. doi: 10.1038/s41467-024-46751-5 (PMC10954709; doi:10.1038/s41467-024-46751-5)
Supplement: Supplementary file 5 — Reporting Summary [file 41467_2024_46751_MOESM5_ESM.pdf]

nature portfolio

Corresponding author(s): Show-Ling Shyng, Camden Driggers

Last updated by author(s): Feb 22, 2024

Reporting Summary

Nature Portfolio wishes to improve the reproducibility of the work that we publish. This form provides structure for consistency and transparency in reporting. For further information on Nature Portfolio policies, see our Editorial Policies and the Editorial Policy Checklist.

Statistics

For all statistical analyses, confirm that the following items are present in the figure legend, table legend, main text, or Methods section.

n/a	Confirmed	
☐	☒	The exact sample size (*n*) for each experimental group/condition, given as a discrete number and unit of measurement
☐	☒	A statement on whether measurements were taken from distinct samples or whether the same sample was measured repeatedly
☐	☒	The statistical test(s) used AND whether they are one- or two-sided<br>*Only common tests should be described solely by name; describe more complex techniques in the Methods section.*
☒	☐	A description of all covariates tested
☐	☒	A description of any assumptions or corrections, such as tests of normality and adjustment for multiple comparisons
☐	☒	A full description of the statistical parameters including central tendency (e.g. means) or other basic estimates (e.g. regression coefficient) AND variation (e.g. standard deviation) or associated estimates of uncertainty (e.g. confidence intervals)
☐	☒	For null hypothesis testing, the test statistic (e.g. *F*, *t*, *r*) with confidence intervals, effect sizes, degrees of freedom and *P* value noted<br>*Give P values as exact values whenever suitable.*
☒	☐	For Bayesian analysis, information on the choice of priors and Markov chain Monte Carlo settings
☒	☐	For hierarchical and complex designs, identification of the appropriate level for tests and full reporting of outcomes
☒	☐	Estimates of effect sizes (e.g. Cohen's *d*, Pearson's *r*), indicating how they were calculated

Our web collection on statistics for biologists contains articles on many of the points above.

Software and code

Policy information about availability of computer code

Data collection	Serial EM
Data analysis	CryoSPARC v4.4.1, UCSF ChimeraX version 1.2, Phenix 1.20.1-4487, Coot 0.9.8.1

For manuscripts utilizing custom algorithms or software that are central to the research but not yet described in published literature, software must be made available to editors and reviewers. We strongly encourage code deposition in a community repository (e.g. GitHub). See the Nature Portfolio guidelines for submitting code & software for further information.

Data

Policy information about availability of data

All manuscripts must include a data availability statement. This statement should provide the following information, where applicable:

- Accession codes, unique identifiers, or web links for publicly available datasets
- A description of any restrictions on data availability
- For clinical datasets or third party data, please ensure that the statement adheres to our policy

TThe cryo-EM maps and structural models of the PIP2-bound open SUR1/Kir6.2Q52R KATP channel have been deposited in the Electron Microscopy Data Bank (EMDB), and the coordinates have been deposited in the PDB under the following accession numbers: PDB ID 8TI2 [https://doi.org/10.2210/pdb8TI2/pdb] and EMD-41278 [https://www.ebi.ac.uk/pdbe/entry/emdb/EMD-41278] (NBD2 modeled as main chain atoms only); PDB ID 8TI1 [https://doi.org/10.2210/pdb8TI1/pdb] and EMD-41277 [https://www.ebi.ac.uk/pdbe/entry/emdb/EMD-41277] (NBD2 not modeled). The cryo-EM map of the apo closed SUR1/Kir6.2Q52R KATP channel

has been deposited in the Electron Microscopy Data Bank under the accession number: EMD-43766 [https://www.ebi.ac.uk/pdbe/entry/emdb/EMD-43766]. Previously published PDB codes referred to in this article include: PDB ID 7UQR [https://doi.org/10.2210/pdb7UQR/pdb] and EMD-26320 [https://www.emdataresource.org/EMD-26320] ; PDB ID 7TYS [https://doi.org/10.2210/pdb7TYS/pdb] and EMD-26193 [https://www.emdataresource.org/EMD-26193]; PDB ID 6BAA [https://doi.org/10.2210/pdb6BAA/pdb] and EMD-7073 [https://www.emdataresource.org/EMD-7073]; [PDB ID 7W4O [https://doi.org/10.2210/pdb7W4O/pdb] and EMD-32310 [https://www.emdataresource.org/EMD-32310]; PDB ID 7S5X [https://doi.org/10.2210/pdb7S5X/pdb] and EMD-24842 [https://www.emdataresource.org/EMD-24842].

Research involving human participants, their data, or biological material

Policy information about studies with human participants or human data. See also policy information about sex, gender (identity/presentation), and sexual orientation and race, ethnicity and racism.

Reporting on sex and gender	N/A
Reporting on race, ethnicity, or other socially relevant groupings	N/A
Population characteristics	N/A
Recruitment	N/A
Ethics oversight	N/A

Note that full information on the approval of the study protocol must also be provided in the manuscript.

Field-specific reporting

Please select the one below that is the best fit for your research. If you are not sure, read the appropriate sections before making your selection.

☒ Life sciences ☐ Behavioural & social sciences ☐ Ecological, evolutionary & environmental sciences

For a reference copy of the document with all sections, see nature.com/documents/nr-reporting-summary-flat.pdf

Life sciences study design

All studies must disclose on these points even when the disclosure is negative.

Sample size	Sample size details for experiments shown in Figures 3 and 7 and Supplementary Figure 7 are described in the figure legends and the Source Data file. The sample size was not predetermined using statistical methods. All functional experiments were repeated at least 3 times to ensure reproducibility of the data, which is standard practice in the field.. The sample sizes are sufficient based on the distribution of data and subsequent statistical analysis of the data.
Data exclusions	No experimental data for experiments shown in Figure 3 and 7 and Supplementary Figure 7 were excluded
Replication	The cryoEM data sample preparation and data collection was performed once. For Rb efflux experiments, two technical replicates were performed in each independent experiments and 3-6 independent experiments were performed. The exact sample size for each experimental group is stated in the figure legends. For electrophysiology experiments, data were collected across 3-5 independent transfections for each channel type. WT channels were always included in each transfection and recorded as a control. The exact number of total cells analyzed for each experimental group is provided in the figure legends and Source Data file. All attempts to replicate the functional experiments were successful.
Randomization	For cryo-EM 3D refinement, all particles were randomly split into two groups. No group allocation was needed for functional experiments in this study.
Blinding	The investigators were blinded to group allocation during cryo-EM half map generation. Blinding is not relevant for protein structure determination and functional assays because these results are not subjective.

Reporting for specific materials, systems and methods

We require information from authors about some types of materials, experimental systems and methods used in many studies. Here, indicate whether each material, system or method listed is relevant to your study. If you are not sure if a list item applies to your research, read the appropriate section before selecting a response.

Materials & experimental systems

n/a	Involved in the study	
☐	☒	Antibodies
☐	☒	Eukaryotic cell lines
☒	☐	Palaeontology and archaeology
☒	☐	Animals and other organisms
☒	☐	Clinical data
☒	☐	Dual use research of concern
☒	☐	Plants

Methods

n/a	Involved in the study	
☒	☐	ChIP-seq
☒	☐	Flow cytometry
☒	☐	MRI-based neuroimaging

Antibodies

Antibodies used: Anti-FLAG M2 affinity gel

Validation: Sigma-Aldrich (A2220)

Eukaryotic cell lines

Policy information about cell lines and Sex and Gender in Research

Cell line source(s): COSm6 (Chlorocebus aethiops; identifier RRID:CVCL_8561); HEK-AD293 cells (RRID:CVCL_0063)

Authentication: COSm6 were not authenticated. HEK-AD293 cells were obtained from from Agilant Technologies catalog number 240085

Mycoplasma contamination: Mycoplasma contamination was ruled out using PCR-based detection kit (checked every 6 months) throughout the duration of the study

Commonly misidentified lines (See ICLAC register): No commonly misidentified cell lines were used in this study
