# Supplementary material for: Structure of an open KATP channel reveals tandem PIP2 binding sites mediating the Kir6.2 and SUR1 regulatory interface
Source: Nat Commun. 2024 Mar 20;15:2502. doi: 10.1038/s41467-024-46751-5 (PMC10954709; doi:10.1038/s41467-024-46751-5)
Supplement: Supplementary file 6 — Source Data [file 41467_2024_46751_MOESM6_ESM.zip › 481233_1_data_set_8715528_s9bsgc.docx]

**Figure 3. Source Data**

| Figure 3b | WT | SUR1^K134A^ | Kir6.2^R176A^ | SUR1^K134A^/Kir6.2^R176A^ |
| --- | --- | --- | --- | --- |
| Initial currents | 612 | 311.2 | 93.1 | 22.55 |
|  | 1748 | 601 | 58 | 19.75 |
|  | 936 | 1636.75 | 54 | 5.5 |
|  | 1484 | 553 | 40 | 24 |
|  | 4304 | 173.5 | 32 | 0.04 |
|  | 3521 | 306 | 6 | 3.86 |
|  | 525.7 |  | 14 |  |
|  | 885 |  |  |  |
|  | 427.6 |  |  |  |
|  | 1358 |  |  |  |
|  | 950 |  |  |  |
|  | 1111 |  |  |  |
| mean | 1488.53 | 596.91 | 42.44 | 12.62 |
| SEM | 348.86 | 218.23 | 11.12 | 4.34 |
| n | 12 | 6 | 7 | 6 |
|  | | | | |
| Figure 3c | WT | SUR1^K134A^ | Kir6.2^R176A^ | SUR1^K134A^/Kir6.2^R176A^ |
| I fold increase | 1.83 | 3.99 | 9.72 | 146.93 |
|  | 3.07 | 4.15 | 20.49 | 656.92 |
|  | 1.03 | 3.72 | 14.15 | 72.47 |
|  | 1.28 | 2.52 | 21.13 | 94.77 |
|  | 0.99 | 5.99 | 143.67 | 527.14 |
|  | 1.11 | 3.35 | 59.17 |  |
|  | 1.80 |  | 72.86 |  |
|  | 0.81 |  |  |  |
|  | 1.59 |  |  |  |
|  | 1.04 |  |  |  |
|  | 1.22 |  |  |  |
|  | 1.64 |  |  |  |
| mean | 1.45 | 3.96 | 48.74 | 299.65 |
| SEM | 0.18 | 0.47 | 18.25 | 121.72 |
| n | 12 | 6 | 7 | 5 |
|  | | | | |
| Figure 3d | WT | SUR1^K134A^ | Kir6.2^R176A^ | SUR1^K134A^/Kir6.2^R176A^ |
| Time to max (sec) | 43 | 189 | 729 | 359 |
|  | 26 | 270 | 183 | 623 |
|  | 17 | 305 | 238 | 331 |
|  | 44 | 185 | 236 | 553 |
|  | 36 | 125 | 280 | 248 |
|  | 43 | 89 | 153 | 402 |
|  | 40 |  | 271 |  |
|  | 23 |  |  |  |
|  | 30 |  |  |  |
|  | 36 |  |  |  |
|  | 46 |  |  |  |
|  | 14 |  |  |  |
| mean | 33.17 | 193.83 | 298.57 | 419.33 |
| SEM | 3.18 | 33.66 | 73.76 | 57.86 |
| n | 12 | 6 | 7 | 6 |

**Figure 7. Source Data**

| Figure 7c | % current | | | | | | |
| --- | --- | --- | --- | --- | --- | --- | --- |
|  | 1 μM | 10 μM | 100 μM | 200 μM | 1 mM | 2 mM | 5 mM |
| WT |  | 81.06 | 17.44 | 5.22 |  | 0.00 |  |
| WT |  |  |  |  | 4.17 |  | 3.15 |
| WT |  | 20.57 | 2.41 | 1.09 |  | 0.86 |  |
| WT |  | 40.00 | 3.92 | 1.69 |  | 0.76 |  |
| WT |  | 45.85 | 11.25 | 7.61 |  | 1.00 |  |
| WT |  | 26.24 | 0.45 | 0.03 |  |  |  |
| WT | 95.62 | 51.98 |  |  | 4.15 |  | 3.05 |
| WT | 77.86 | 36.29 |  |  | 4.19 |  | 2.59 |
| WT | 80.85 | 11.07 |  |  |  |  |  |
| mean | 84.78 | 39.13 | 7.09 | 3.13 | 4.17 | 0.65 | 2.98 |
| SEM | 5.49 | 7.65 | 3.17 | 1.42 | 0 | 0.22 | 0.17 |
| n | 3 | 8 | 5 | 5 | 3 | 4 | 3 |
|  | | | | | | | |
|  | 1 μM | 10 μM | 100 μM | 200 μM | 1 mM | 2 mM | 5 mM |
| Kir6.2^Q52R^ |  |  |  |  | 21.99 |  | 4.84 |
| Kir6.2^Q52R^ |  |  | 96.00 |  | 15.51 |  | 0.91 |
| Kir6.2^Q52R^ |  | 97.69 | 96.64 | 11.06 |  | 5.80 |  |
| Kir6.2^Q52R^ |  | 71.29 | 65.69 | 4.29 |  | 3.07 |  |
| Kir6.2^Q52R^ |  | 98.93 | 84.51 | 37.70 |  | 1.95 |  |
| Kir6.2^Q52R^ |  | 98.64 | 97.00 | 90.33 |  | 1.64 |  |
| Kir6.2^Q52R^ |  | 100.04 | 72.27 | 43.80 |  | 0.93 |  |
| Kir6.2^Q52R^ |  | 94.04 | 32.13 | 9.87 |  | 0.15 |  |
| Kir6.2^Q52R^ |  | 99.72 | 79.52 | 46.37 |  | 1.30 |  |
| Kir6.2^Q52R^ |  |  |  |  | 7.74 |  | 2.88 |
| mean |  | 94.34 | 77.97 | 34.77 | 15.08 | 2.12 | 2.88 |
| SEM |  | 3.91 | 7.75 | 11.36 | 4.12 | 0.70 | 1.13 |
| n |  | 7 | 8 | 7 | 3 | 7 | 3 |
|  | | | | | | | |
|  | 1 μM | 10 μM | 100 μM | 200 μM | 1 mM | 2 mM | 5 mM |
| SUR1^W51C^ |  | 62.52 | 5.12 | 3.42 |  | 1.10 |  |
| SUR1^W51C^ |  | 64.98 | 6.72 | 6.44 |  | 0.84 |  |
| SUR1^W51C^ |  | 97.05 | 21.57 | 6.56 |  | 0.08 |  |
| SUR1^W51C^ |  |  |  | 14.89 |  | 13.10 |  |
| SUR1^W51C^ |  | 79.14 | 0.65 | 0.49 |  | 0.27 |  |
| SUR1^W51C^ | 85.71 |  | 0.36 |  | 0.21 |  | 0.01 |
| SUR1^W51C^ | 104.58 |  | 4.22 |  | 2.22 |  | -0.00 |
| SUR1^W51C^ | 94.38 |  | 4.78 |  | 3.35 |  | 0.00 |
| mean | 94.89 | 75.92 | 6.20 | 6.36 | 1.93 | 3.08 | 0 |
| SEM | 5.45 | 7.94 | 2.71 | 2.41 | 0.92 | 2.51 | 0 |
| n | 3 | 4 | 7 | 5 | 3 | 5 | 3 |
|  | | | | | | | |
|  | 1 μM | 10 μM | 100 μM | 200 μM | 1 mM | 2 mM | 5 mM |
| SUR1^W51C^/Kir6.2^Q52R^ |  |  |  |  | 4.17 |  | 3.15 |
| SUR1^W51C^/Kir6.2^Q52R^ |  |  | 11.13 |  | 0.69 |  | 0.09 |
| SUR1^W51C^/Kir6.2^Q52R^ |  |  | 9.76 |  | 4.91 |  | 3.27 |
| SUR1^W51C^/Kir6.2^Q52R^ |  |  | 2.21 |  | 1.30 |  | 0.76 |
| SUR1^W51C^/Kir6.2^Q52R^ |  | 35.27 | 3.02 | 0.64 |  | 0.22 |  |
| SUR1^W51C^/Kir6.2^Q52R^ |  | 32.25 | 5.92 | 1.83 |  | 0.85 |  |
| SUR1^W51C^/Kir6.2^Q52R^ |  | 14.68 |  |  | 2.45 | 0.79 |  |
| SUR1^W51C^/Kir6.2^Q52R^ | 87.56 | 27.12 |  | 1.25 | 0.00 |  |  |
| SUR1^W51C^/Kir6.2^Q52R^ |  | 37.15 |  |  | 0.80 |  |  |
| SUR1^W51C^/Kir6.2^Q52R^ | 109.61 | 89.30 |  |  | 0.00 |  |  |
| SUR1^W51C^/Kir6.2^Q52R^ | 95.58 | 82.76 |  |  | 3.57 |  |  |
| mean | 97.59 | 45.50 | 6.41 | 0.85 | 1.99 | 0.62 | 1.82 |
| SEM | 6.44 | 10.85 | 1.77 | 0.51 | 0.62 | 0.2 | 0.81 |
| n | 3 | 7 | 5 | 3 | 9 | 3 | 4 |

| Figure 7d | % Rb^+^ efflux | | | | |
| --- | --- | --- | --- | --- | --- |
|  | Untransfected | WT | SUR1^W51C^ | Kir6.2^Q52R^ | SUR1^W51C^/ Kir6.2^Q52R^ |
| Exp 1 | 43.57 | 45.63 | 42.25 | 81.97 | 42.55 |
| Exp 2 | 43.04 | 46.81 | 41.56 | 79.71 | 42.35 |
| Exp 3 | 46.06 | 62.56 | 47.43 | 90.27 | 59.23 |
| Exp 4 | 47.83 | 73.90 | 52.72 | 93.01 | 53.85 |
| Exp 5 | 47.67 | 58.20 | 50.20 | 92.55 | 49.31 |
| mean | 45.63 | 57.42 | 46.83 | 87.50 | 49.46 |
| SEM | 1.00 | 5.25 | 2.18 | 2.78 | 3.26 |
| n | 5 | 5 | 5 | 5 | 5 |

**Supplementary Figure 7a. Source Data**

*Note data for WT and Kir6.2^Q52R^ in Supplementary Figure 7a are the same as in Figure 7c.

|  | % current | | | | | | |
| --- | --- | --- | --- | --- | --- | --- | --- |
|  | 1 μM | 10 μM | 100 μM | 200 μM | 1 mM | 2 mM | 5 mM |
| WT |  | 81.06 | 17.44 | 5.22 |  | 0.00 |  |
| WT |  |  |  |  | 4.17 |  | 3.15 |
| WT |  | 20.57 | 2.41 | 1.09 |  | 0.86 |  |
| WT |  | 40.00 | 3.92 | 1.69 |  | 0.76 |  |
| WT |  | 45.85 | 11.25 | 7.61 |  | 1.00 |  |
| WT |  | 26.24 | 0.45 | 0.03 |  |  |  |
| WT | 95.62 | 51.98 |  |  | 4.15 |  | 3.05 |
| WT | 77.86 | 36.29 |  |  | 4.19 |  | 2.59 |
| WT | 80.85 | 11.07 |  |  |  |  |  |
| mean | 84.78 | 39.13 | 7.09 | 3.13 | 4.17 | 0.65 | 2.98 |
| SEM | 5.49 | 7.65 | 3.17 | 1.42 | 0 | 0.22 | 0.17 |
| n | 3 | 8 | 5 | 5 | 3 | 4 | 3 |
|  | | | | | | | |
|  | 1 μM | 10 μM | 100 μM | 200 μM | 1 mM | 2 mM | 5 mM |
| Kir6.2^Q52R^ |  |  |  |  | 21.99 |  | 4.84 |
| Kir6.2^Q52R^ |  |  | 96.00 |  | 15.51 |  | 0.91 |
| Kir6.2^Q52R^ |  | 97.69 | 96.64 | 11.06 |  | 5.80 |  |
| Kir6.2^Q52R^ |  | 71.29 | 65.69 | 4.29 |  | 3.07 |  |
| Kir6.2^Q52R^ |  | 98.93 | 84.51 | 37.70 |  | 1.95 |  |
| Kir6.2^Q52R^ |  | 98.64 | 97.00 | 90.33 |  | 1.64 |  |
| Kir6.2^Q52R^ |  | 100.04 | 72.27 | 43.80 |  | 0.93 |  |
| Kir6.2^Q52R^ |  | 94.04 | 32.13 | 9.87 |  | 0.15 |  |
| Kir6.2^Q52R^ |  | 99.72 | 79.52 | 46.37 |  | 1.30 |  |
| Kir6.2^Q52R^ |  |  |  |  | 7.74 |  | 2.88 |
| mean |  | 94.34 | 77.97 | 34.77 | 15.08 | 2.12 | 2.88 |
| SEM |  | 3.91 | 7.75 | 11.36 | 4.12 | 0.70 | 1.13 |
| n |  | 7 | 8 | 7 | 3 | 7 | 3 |
|  | | | | | | | |
|  | 1 μM | 10 μM | 100 μM | 200 μM | 1 mM | 2 mM | 5 mM |
| SUR1^K134A^ | 99.06 |  | 20.19 |  | 3.77 |  |  |
| SUR1^K134A^ | 74.65 |  | 0.11 |  | -0.21 |  |  |
| SUR1^K134A^ | 91.40 |  | 0.43 |  | 0.10 |  |  |
| SUR1^K134A^ | 96.00 |  | 0.60 |  | 0.04 |  |  |
| SUR1^K134A^ |  | 15.94 |  | 0.46 |  | 0.22 |  |
| SUR1^K134A^ |  | 29.72 |  | 0.79 |  | 0.01 |  |
| SUR1^K134A^ |  | 13.76 |  | 1.27 |  | 0.51 |  |
| mean | 90.28 | 19.80 | 5.33 | 0.84 | 0.93 | 0.25 |  |
| SEM | 5.44 | 5.00 | 4.95 | 0.24 | 0.95 | 0.14 |  |
| n | 4 | 3 | 4 | 3 | 4 | 3 |  |
|  | | | | | | | |
|  | 1 μM | 10 μM | 100 μM | 200 μM | 1 mM | 2 mM | 5 mM |
| SUR1^K134A^/Kir6.2^Q52R^ |  |  | 23.86 | 2.60 |  |  | 0.21 |
| SUR1^K134A^/Kir6.2^Q52R^ |  | 91.83 | 25.64 | 6.95 |  |  | 0.23 |
| SUR1^K134A^/Kir6.2^Q52R^ |  | 92.21 | 30.76 | 9.45 |  |  | 0.00 |
| SUR1^K134A^/Kir6.2^Q52R^ |  | 86.35 | 11.07 | 2.61 |  |  | 0.00 |
| SUR1^K134A^/Kir6.2^Q52R^ | 96.63 |  | 57.81 |  | 1.49 |  | 0 |
| SUR1^K134A^/Kir6.2^Q52R^ | 99.82 |  | 59.95 |  | 0.86 |  | 0.00 |
| SUR1^K134A^/Kir6.2^Q52R^ | 98.81 |  | 15.55 |  | 0.04 |  | 0.00 |
| mean | 98.42 | 90.13 | 32.09 | 5.40 | 0.80 |  | 0.06 |
| SEM | 0.94 | 1.89 | 7.34 | 1.69 | 0.42 |  | 0.04 |
| n | 3 | 3 | 4 | 4 | 3 |  | 7 |
|  | | | | | | | |
|  | 1 μM | 10 μM | 100 μM | 200 μM | 1 mM | 2 mM | 5 mM |
| Kir6.2^R176A^ | 90.14 | 81.33 | 5.42 |  |  | 1.61 |  |
| Kir6.2^R176A^ | 74.66 | 37.92 | 7.66 |  |  | 0.79 |  |
| Kir6.2^R176A^ | 106.82 | 56.25 | 11.36 |  |  | 2.84 |  |
| Kir6.2^R176A^ | 62.16 | 69.82 | 5.41 |  |  | 0.68 |  |
| Kir6.2^R176A^ |  | 41.99 | 6.30 |  | 2.27 |  |  |
| Kir6.2^R176A^ |  | 54.53 | 11.47 |  | 9.77 |  |  |
| mean | 83.45 | 56.97 | 7.94 |  | 6.02 | 1.48 |  |
| SEM | 9.67 | 6.71 | 1.15 |  | 3.75 | 0.50 |  |
| n | 4 | 6 | 6 |  | 3 | 4 |  |
|  | | | | | | | |
|  | 1 μM | 10 μM | 100 μM | 200 μM | 1 mM | 2 mM | 5 mM |
| Kir6.2^Q52R/R176A^ |  |  | 4.39 |  | -0.86 |  |  |
| Kir6.2^Q52R/R176A^ |  |  | 8.20 |  | 0.36 |  |  |
| Kir6.2^Q52R/R176A^ |  | 89.34 | 5.75 |  | 1.96 |  |  |
| Kir6.2^Q52R/R176A^ |  |  | 9.19 |  | 2.21 |  |  |
| Kir6.2^Q52R/R176A^ | 94.43 | 70.10 |  | 0.79 |  | 0.06 |  |
| Kir6.2^Q52R/R176A^ | 93.78 | 63.43 |  | 0.54 |  | 0.18 |  |
| Kir6.2^Q52R/R176A^ | 91.24 | 62.15 |  | 1.22 |  | 0.16 |  |
| Kir6.2^Q52R/R176A^ | 98.20 | 51.68 |  | 7.18 |  | 0.05 |  |
| Kir6.2^Q52R/R176A^ | 90.58 | 85.37 |  | 7.79 |  | 0.13 |  |
| mean | 93.65 | 70.34 | 6.88 | 3.51 | 0.92 | 0.12 |  |
| SEM | 1.35 | 5.92 | 1.10 | 1.63 | 0.65 | 0.03 |  |
| n | 5 | 6 | 4 | 5 | 5 | 5 |  |
|  |  |  |  |  |  |  |  |
|  | 1 μM | 10 μM | 100 μM | 200 μM | 1 mM | 2 mM | 5 mM |
| SUR1^K134A^/Kir6.2^Q52R/R176A^ | 40.58 | 16.08 | 8.65 |  |  | 6.87 |  |
| SUR1^K134A^/Kir6.2^Q52R/R176A^ |  | 37.01 | 1.27 |  | 0.68 |  | -0.01 |
| SUR1^K134A^/Kir6.2^Q52R/R176A^ |  |  | 1.60 |  | -0.62 |  | 0.00 |
| SUR1^K134A^/Kir6.2^Q52R/R176A^ |  | 36.25 | 2.92 |  | -1.28 |  | 0.02 |
| SUR1^K134A^/Kir6.2^Q52R/R176A^ |  |  | 8.45 |  | 0.56 |  | 0.00 |
| SUR1^K134A^/Kir6.2^Q52R/R176A^ |  | 77.14 | 11.13 |  | 0.35 |  |  |
| SUR1^K134A^/Kir6.2^Q52R/R176A^ |  | 45.81 | 1.17 |  | 0.15 |  |  |
| SUR1^K134A^/Kir6.2^Q52R/R176A^ |  | 55.12 | 1.48 |  | -0.04 |  |  |
| mean | 40.58 | 44.57 | 4.58 |  | -0.08 | 6.97 | 0.00 |
| SEM | - | 8.39 | 1.45 |  | 0.26 | - | 0.00 |
| n | 1 | 6 | 8 |  | 7 | 1 | 4 |

**Supplementary Figure 7b source data**

|  | % Rb^+^ efflux | | | | | | | | | |
| --- | --- | --- | --- | --- | --- | --- | --- | --- | --- | --- |
|  | Untransfected | | | WT | SUR1^K134A^ | | Kir6.2^R176A^ | | SUR1^K134A^/Kir6.2^R176A^ | |
| Exp 1 | 28.09 | | | 39.05 | 32.48 | | 33.03 | |  | |
| Exp 2 | 32.01 | | | 34.11 | 30.52 | | 33.02 | |  | |
| Exp 3 | 27.75 | | | 35.19 | 31.86 | | 28.59 | |  | |
| Exp 4 | 27.39 | | | 39.59 | 35.99 | | 31.06 | | 36.53 | |
| Exp 5 | 28.62 | | | 38.81 | 35.97 | | 30.95 | | 35.27 | |
| Exp 6 | 27.67 | | | 40.94 | 33.12 | | 32.05 | | 33.79 | |
| mean | 28.59 | | | 37.95 | 33.32 | | 31.45 | | 35.20 | |
| SEM | 0.71 | | | 1.09 | 0.91 | | 0.68 | | 0.79 | |
| n | 6 | | | 6 | 6 | | 6 | | 3 | |
|  |  | | |  |  | |  | |  | |
|  |  | | |  |  | |  | |  | |
|  |  | | |  |  | |  | |  | |
|  | | Kir6.2^Q52R^ | SUR1^K134A^/Kir6.2^Q52R^ | | | Kir6.2^Q52R/R176A^ | | SUR1^K134A^/Kir6.2^Q52R/R176A^ | |  |
| Exp 1 | | 84.13 | 71.69 | | | 64.21 | |  | |  |
| Exp 2 | | 70.59 | 58.44 | | | 52.13 | |  | |  |
| Exp 3 | | 68.97 | 56.55 | | | 51.54 | |  | |  |
| Exp 4 | | 80.21 | 72.28 | | | 57.58 | | 44.82 | |  |
| Exp 5 | | 80.47 | 75.40 | | | 60.08 | | 44.40 | |  |
| Exp 6 | | 79.95 | 75.03 | | | 63.05 | | 42.69 | |  |
| mean | | 77.39 | 68.23 | | | 58.10 | | 43.97 | |  |
| SEM | | 2.49 | 3.46 | | | 2.20 | | 0.65 | |  |
| n | | 6 | 6 | | | 6 | | 3 | |  |
